# Supplementary material for: Postnatal corticosteroid use for prevention or treatment of bronchopulmonary dysplasia in England and Wales 2012–2019: a retrospective population cohort study
Source: BMJ Open. 2022 Nov 16;12(11):e063835. doi: 10.1136/bmjopen-2022-063835 (PMC9676997; doi:10.1136/bmjopen-2022-063835)
Supplement: Supplementary data [file bmjopen-2022-063835supp001.pdf]

Supplementary file S1

Code	Diagnosis
15817	Neonatal intestinal perforation
15819	Neonatal ileum perforation
15821	Neonatal terminal ileum perforation
11010244	Intestinal perforation - not NEC
11217	Closure of jejunal perforation
11222	Closure of small intestine/ileal perforation
