# Supplementary material for: Postnatal corticosteroid use for prevention or treatment of bronchopulmonary dysplasia in England and Wales 2012–2019: a retrospective population cohort study
Source: BMJ Open. 2022 Nov 16;12(11):e063835. doi: 10.1136/bmjopen-2022-063835 (PMC9676997; doi:10.1136/bmjopen-2022-063835)
Supplement: Author's manuscript [file bmjopen-2022-063835.draft_revisions.pdf]

BMJ Open

BMJ Open is committed to open peer review. As part of this commitment we make the peer review history of every article we publish publicly available.

When an article is published we post the peer reviewers' comments and the authors' responses online. We also post the versions of the paper that were used during peer review. These are the versions that the peer review comments apply to.

The versions of the paper that follow are the versions that were submitted during the peer review process. They are not the versions of record or the final published versions. They should not be cited or distributed as the published version of this manuscript.

BMJ Open is an open access journal and the full, final, typeset and author-corrected version of record of the manuscript is available on our site with no access controls, subscription charges or pay-per-view fees (http://bmjopen.bmj.com).

If you have any questions on BMJ Open's open peer review process please email info.bmjopen@bmj.com

BMJ Open

Postnatal corticosteroid use for prevention or treatment of Bronchopulmonary Dysplasia in England and Wales 2012-2019: a retrospective population cohort study

Journal:	*BMJ Open*
Manuscript ID	bmjopen-2022-063835
Article Type:	Original research
Date Submitted by the Author:	13-Apr-2022
Complete List of Authors:	Yao, Sijia; Imperial College London, Neonatal Medicine<br>Uthaya, Sabita; Imperial College London, Neonatal Medicine, School of Public Health<br>Gale, Chris; Imperial College London, Neonatal Medicine, School of Public Health<br>Modi, Neena; Imperial College London, Neonatal Medicine, School of Public Health<br>Battersby, Cheryl; Imperial College London, Neonatal Medicine, School of Public Health
Keywords:	NEONATOLOGY, Neonatal intensive & critical care < INTENSIVE & CRITICAL CARE, RESPIRATORY MEDICINE (see Thoracic Medicine)

SCHOLARONE™
Manuscripts

I, the Submitting Author has the right to grant and does grant on behalf of all authors of the Work (as defined in the below author licence), an exclusive licence and/or a non-exclusive licence for contributions from authors who are: i) UK Crown employees; ii) where BMJ has agreed a CC-BY licence shall apply, and/or iii) in accordance with the terms applicable for US Federal Government officers or employees acting as part of their official duties; on a worldwide, perpetual, irrevocable, royalty-free basis to BMJ Publishing Group Ltd ("BMJ") its licensees and where the relevant Journal is co-owned by BMJ to the co-owners of the Journal, to publish the Work in this journal and any other BMJ products and to exploit all rights, as set out in our licence.

The Submitting Author accepts and understands that any supply made under these terms is made by BMJ to the Submitting Author unless you are acting as an employee on behalf of your employer or a postgraduate student of an affiliated institution which is paying any applicable article publishing charge ("APC") for Open Access articles. Where the Submitting Author wishes to make the Work available on an Open Access basis (and intends to pay the relevant APC), the terms of reuse of such Open Access shall be governed by a Creative Commons licence – details of these licences and which Creative Commons licence will apply to this Work are set out in our licence referred to above.

Other than as permitted in any relevant BMJ Author's Self Archiving Policies, I confirm this Work has not been accepted for publication elsewhere, is not being considered for publication elsewhere and does not duplicate material already published. I confirm all authors consent to publication of this Work and authorise the granting of this licence.

For peer review only

Title:

Postnatal corticosteroid use for prevention or treatment of Bronchopulmonary Dysplasia in England and Wales 2012-2019: a retrospective population cohort study

Authors

S Yao[1], S Uthaya[1,2], C Gale[1,2], N Modi[1,2], C Battersby[1,2] and the UK Neonatal Collaborative*

1. Neonatal Medicine, School of Public Health, Faculty of Medicine, Imperial College London, London, United Kingdom
2. Chelsea and Westminster NHS Foundation Trust

List of neonatal units*

Corresponding author

Cheryl Battersby, PhD

Section of Neonatal Medicine, Chelsea and Westminster campus, Imperial College London. 369 Fulham Road, London, SW10 9NH, United Kingdom.

c.battersby@imperial.ac.uk

Keywords

Neonatal; postnatal corticosteroids; preterm; bronchopulmonary dysplasia

2049/2500

For peer review only

Abstract

Objective: Describe the population of babies who do and do not receive postnatal corticosteroids for prevention or treatment of BPD

Design: Retrospective cohort study using data held in the National Neonatal Research Database

Setting: National Health Service neonatal units in England and Wales

Patients: Babies born less than 32 weeks gestation and admitted to neonatal units from 1st January 2012 to 31st December 2019.

Main outcomes: Proportion of babies given postnatal corticosteroid; type of corticosteroid; age at initiation and duration, trends over time

Secondary outcomes: Survival to discharge, treatment for retinopathy of prematurity, bronchopulmonary dysplasia, brain injury, severe necrotising enterocolitis, gastrointestinal perforation.

Results: 8% (4713/62019) of babies born <32 weeks and 26% (3525/13527) born <27 weeks received postnatal corticosteroids for BPD. Dexamethasone was predominantly used 5.3% (3309/62019), followed by late hydrocortisone 1.5%, inhaled budesonide 1.5%. prednisolone 0.8%, early hydrocortisone 0.3% and methylprednisolone 0.05%. Dexamethasone use increased over time (2012: 4.5 vs. 2019: 5.8%, p=0.04). Median postnatal age of initiation of corticosteroid course was around 3 weeks for *late hydrocortisone*, 4 weeks for dexamethasone, 6 weeks for inhaled budesonide,12 weeks for prednisolone and 16 weeks for methylprednisolone. Babies who received postnatal corticosteroids were born more prematurely, had a higher incidence of comorbidities and a longer length of stay.

Conclusions: In England and Wales, around 1 in 12 babies born less than 32 weeks and 1 in 4 born less than 27 weeks receive postnatal corticosteroids to prevent or treat bronchopulmonary dysplasia. Given the lack of convincing evidence of efficacy, challenges of recruiting to and length of time taken to conduct RCT, our data highlight the need to monitor long-term outcomes in children who received neonatal postnatal corticosteroids.

Strengths and limitations of this study

Data for sixty-two thousand and nineteen preterm babies were included in this study

For peer review only - http://bmjopen.bmj.com/site/about/guidelines.xhtml

Exposure to different types of postnatal corticosteroids, including timing of initiation and duration are described

Data over an eight-year period enabled time trends to be evaluated

Lack of data on the indication for postnatal corticosteroid may lead to overestimation of babies exposed to hydrocortisone for BPD versus for blood pressure or adrenal insufficiency

We were unable to report cumulative doses of dexamethasone as this was not available

Patient and Public Involvement statement

Patients or the public were not involved in the design, or conduct, or reporting, or dissemination plans of our research

Introduction

Bronchopulmonary dysplasia (BPD), defined as needing respiratory support at 36 weeks post-menstrual age, is the most common adverse outcome of very preterm delivery, affecting up to 75% of babies born before 28 weeks of gestation worldwide (1, 2). In the most severely affected, supplemental oxygen at home is needed, pulmonary hypertension may develop and lung-function abnormalities can persist into adulthood. BPD is also associated with delayed brain maturation and diffuse white matter anomalies that are associated with increased risk of neurodevelopmental impairment (3). Despite increased use of less invasive ventilation after birth, there has been no significant decline in BPD rates globally, and nor improvement in lung function in childhood over time (4). The pathogenesis of BPD remains to be fully understood but involves arrest of lung development (alveolar hypoplasia or dysmorphic pulmonary vasculature) and pulmonary inflammation. It is therefore postulated that the anti-inflammatory action of postnatal corticosteroids (PNC) may improve gas exchange and lung mechanics, thus facilitating weaning from invasive mechanical ventilation and improving respiratory outcomes (5). However, the use of PNC remains controversial because of fear of long-term adverse neurodevelopmental effects, hypertension, intestinal perforation and impaired glucose metabolism (6-8). Whilst early use of dexamethasone (<7 days) is associated with increased incidence of cerebral palsy (9), there is no strong evidence from available studies to suggest that later (>7 days) administration of lower dose dexamethasone, or early prophylactic use of low dose hydrocortisone, causes neurodevelopmental impairment (10, 11).

A recent systematic review and network meta-analysis included 62 studies involving 5559 neonates with a mean gestational age of 26 weeks and examined 14 different postnatal

For peer review only - http://bmjopen.bmj.com/site/about/guidelines.xhtml

corticosteroid regimens. This found that a moderately early-initiated (8-14 days), medium cumulative dose of systemic dexamethasone (2-4mg/kg) was the best regimen for preventing mortality or BPD but acknowledged the weak evidence upon which this recommendation is based (12). Hence, when considering PNC treatment clinicians have to balance the adverse consequences of long-term ventilation with the potential for long-term neurodisability.

International studies report varying use of PNC (3%-50%) including both systemic and inhaled PNC for preterm neonates (13, 14). Policy recommendations regarding the use of dexamethasone or other corticosteroids, including hydrocortisone and inhaled corticosteroids differ widely (15-17). We aimed to describe the use of PNC for prevention or treatment of BPD over an eight year period in very preterm babies born 2012-2019 in England and Wales, using the National Neonatal Research Database (NNRD). Objectives: i) to quantify the number and proportion of babies who received PNC, timing and duration ii) to describe the clinical characteristics of babies and unadjusted outcomes for babies who do and do not receive PNC

Methods

We undertook a retrospective and descriptive cohort study using deidentified, routinely recorded neonatal clinical data held in the NNRD. The NNRD is a national resource containing detailed, quality assured, clinical information extracted from the electronic patient records of admissions to NHS neonatal units (18, 19). The study is covered by Research Ethics Committee Approval reference 16/LO/1093.

Study population

We included data held in the NNRD on all babies born less than 32 weeks gestation born and admitted to neonatal care units in England and Wales between 1st January 2012 and 31st December 2019. We excluded babies with missing gestational ages. We extracted variables related to maternal factors (ethnicity, age, antenatal steroids); infant demographics (birth weight, sex, gestational age), clinical characteristics (condition at birth including Apgar score and resuscitation details); postnatal corticosteroid use including methylprednisolone, prednisolone, hydrocortisone, dexamethasone, budesonide; respiratory support, co-morbidities (gastrointestinal perforation, bronchopulmonary dysplasia, retinopathy of prematurity that required treatment, brain injury on imaging) (20, 21) and survival to discharge from neonatal care. Data were extracted using SAS version 13.

For peer review only - http://bmjopen.bmj.com/site/about/guidelines.xhtml

Definitions

Postnatal corticosteroids may be used for indications other than prevention or treatment of BPD (upper airway obstruction, blood pressure); however indication for postnatal steroid use is not held in the NNRD. We therefore applied the following pragmatic definitions to identify babies we considered to have received postnatal steroids for BPD prevention or treatment:

Dexamethasone: Dexamethasone for ≥ 5 consecutive days (5 days was chosen to avoid the inclusion of babies given a shorter course for upper airway obstruction)
Early hydrocortisone: hydrocortisone started on postnatal day 1 or 2 and given for >7 consecutive days consistent with the PREMILOC trial (22).
Late hydrocortisone: hydrocortisone started after postnatal day 2 and given for >2 consecutive and >7 days total duration (to differentiate this from hydrocortisone given for hypotension)

Prednisolone: prednisolone given for ≥ 3 consecutive days

Methylprednisolone: methylprednisolone given for ≥ 3 consecutive days

Inhaled budesonide: Use of inhaled budesonide for ≥ 1 day

Bronchopulmonary dysplasia was defined as receiving any respiratory or ventilatory support or supplemental oxygen at 36 weeks postmenstrual age (23) , treatment for retinopathy of prematurity (ROP) was defined as cryotherapy, laser therapy or injection of anti-vascular endothelial growth factor therapy for ROP in either or both eyes, severe necrotising enterocolitis was defined as necrotising enterocolitis resulting in surgery or confirmed at surgery (24), brain injury was defined as either left or right grade 3 or higher intraventricular haemorrhage or periventricular leukomalacia (20), gastrointestinal perforation was defined as having a perforation if presence of relevant diagnostic and/or procedural codes (Supplemental file S1).

Data analysis

Simple descriptive statistics were used to describe the study population, clinical characteristics, survival and co-morbidities without adjustment. We report results for the entire cohort of babies born <32 weeks and also a subgroup of the most preterm babies born <27 weeks gestation based on the British Association of Perinatal Medicine (BAPM) extreme preterm criteria (25). Data were described using medians and interquartile ranges for continuous and non-parametric (as determined by a Shapiro-Wilk normality test) data,

For peer review only - http://bmjopen.bmj.com/site/about/guidelines.xhtml

and proportions for categorical data. Temporal trends were assessed using linear regression. For all the statistical results, a p value <0.05 was considered as statistically significant. Analyses were performed using RStudio, SPSS, and GraphPad Prism 5.

Results

Number and proportion of babies over time who received postnatal corticosteroids for BPD

A total of 62,019 babies born <32 weeks gestation were born and admitted to neonatal units in England and Wales during the study period. 8140 (13%) babies were recorded as having received PNC for any indication and 7.6% (4713/62019) received at least one type of PNC for BPD (Figure 1). The most commonly used PNC was *dexamethasone* 5.3% (3309/62019), followed by *late hydrocortisone* 1.5% (954/62019), *inhaled budesonide* 1.5% (922/62019), *prednisolone* 0.8% (500/62019). *Early hydrocortisone* 0.3% (180/62019) and *methylprednisolone* 0.05% (32/62019) use were rare. 6.3% (3914/62019) received only one type of PNC (*early hydrocortisone* 156, *late hydrocortisone* 620, *dexamethasone* 2461, *prednisolone* 196, *inhaled budesonide* 481). 1.3% (799/62019) received more than one type of PNC.

Between 2012 and 2019, an increasing proportion of very preterm babies received PNC (p=0.005) (Figure 2). There was an overall increase use over time for *dexamethasone*, *inhaled budesonide* and *late hydrocortisone* even though there was a small decrease between 2018 and 2019; *dexamethasone* (2012: 4.5% vs. 2018: 6.6% vs. 2019: 5.8%, 2.0% increase per year, p=0.04); *inhaled budesonide* (2012: 0.6% vs.2018: 2.5% vs. 2019: 2.4%, 19.3% increase per year, p<0.001) (922 babies); *late hydrocortisone* (2012: 1.2% vs. 2018: 2.0% vs. 2019: 1.7%, 4.1% increase per year, p =0.02). The number for *early hydrocortisone* was very small (180) and its use was first seen in 2017 and has increased over time (2012: 0.1% vs. 2019: 0.8%, 3.7% increase per year, p=0.05). Use of *prednisolone* has decreased (2012: 1.0% vs. 2019: 0.6%, 7.8% decrease per year, p<0.09) (500 babies) and *methylprednisolone* remained similar over time (2012: 0.0% vs. 2019: 0.1%).

Clinical characteristics of babies overall and by type of postnatal corticosteroid for BPD

The descriptive and clinical characteristics of babies by type of PNC are presented in Table 1. The median (IQR) gestational age for babies who received PNC was 25 (24-27) weeks; 71% received a complete course of antenatal steroids; 87% were intubated at birth. At the

For peer review only

time of PNC initiation, 75% and 20% were receiving invasive and non-invasive ventilatory support, respectively, with the exception of inhaled budesonide where 49% were on invasive ventilation; 7% received drugs for resuscitation or cardiac massage at delivery.

Table 1. Clinical characteristics of infants by type of postnatal corticosteroid <32 weeks gestation

	Dexamethasone (n=3309)	*Late Hydrocortisone (n=954)*	*Prednisolone (n=500)*	*Methyl-prednisolone (n=32)*	*Inhaled Budesonide (n=922)*	*Early hydrocortisone (N=180*
Gestational age in weeks, median (IQR)	25.1 (24.3, 26.6)	25.4 (24.3, 27.6)	25.4 (24.5, 26.7)	24.9 (24.1, 28.0)	25.7 (24.6, 27.3)	25.0 (24.0, 27.0)
Antenatal corticosteroids						
None	166 (5%)	43 (5%)	26 (5%)	2 (6%)	25 (3%)	3 (2%)
Complete	2306 (70%)	677 (71%)	344 (69%)	24 (75%)	699 (76%)	124 (67%)
Incomplete	574 (17%)	165 (17%)	98 (20%)	5 (16%)	151 (16%)	37 (32%)
Missing	2405 (8%)	667 (7%)	32 (6%)	1 (3%)	46 (5%)	-
Birthweight (g) median (IQR)	710 (620, 840)	700 (600, 854)	710 (615, 84)	755 (597, 869)	758 (640, 910)	757 (645, 916)
Sex Male	1975(60%)	591 (62%)	288 (58%)	22 (69%)	580 (61%)	93 (50%)
Apgar 5 mins						
≤ 5	839 (25%)	231 (24%)	115 (23%)	9 (28%)	218 (24%)	61 (33%)
>5	1960 (59%)	602 (63%)	291 (58%)	20 (63%)	621 (67%)	100 (54%)
Missing	510 (15%)	121 (13%)	94 (19%)	3 (9%)	83 (9%)	23 (13%)
Cardiac massage at delivery	245 (7%)	64 (7%)	31 (6%)	3 (9%)	52 (6%)	18 (10%)
Drugs for resuscitation at delivery	255 (8%)	72 (8%)	50 (10%)	2 (6%)	50 (5%)	20 (11%)

For peer review only - http://bmjopen.bmj.com/site/about/guidelines.xhtml

For peer review only

Intubation at delivery	3021 (91%)	838 (88%)	448 (90%)	28 (88%)	698 (76%)	162 (88%)
Postnatal age (days) at initiation of postnatal corticosteroid, median (IQR)	28 (19, 45)	19 (8, 40)	86 (73, 101)	116 (90, 155)	42 (23, 70)	1 (1, 2)
Duration (days), median (IQR)	14 (10, 25)	20 (11, 38)	9 (8, 30)	7 (4, 11)	19 (9,34)	11(10, 14)
Respiratory support at time of steroid initiation<br>*Invasive ventilation*<br>*Non-invasive support*<br>*None of the above*	2799 (85%)<br>445 (13%)<br>64 (2%)	718 (75%)<br>145 (18%)<br>90 (9%)	372 (74%)<br>86 (17%)<br>42 (8%)	26 (81%)<br>4 (13%)<br>2 (6%)	448 (49%)<br>422 (46%)<br>51 (6%)	171 (93%)<br>12 (7%)<br>1 (0.5%)

For peer review only

Timing and duration of postnatal corticosteroids for BPD

Median (IQR) postnatal age at initiation was 19 (8-40) days for *late hydrocortisone*, 28 (19-45) days for *dexamethasone*, 42 (30-70) days for *inhaled budesonide*, 865 (73-101) for *prednisolone* and 116 (90-155) days for methylprednisolone (Table 1). Over time, the postnatal age at initiation for all PNC remained fairly consistent except for *inhaled budesonide* which is being used earlier in recent years (2012 median (IQR) 79 (57,116) vs 2019 30 (14-51)) (Figure 3). The duration of use was shortest for *methylprednisolone* 7 (4-11) days and longest for *late hydrocortisone* 20 (11-38) days (Table 1).

For peer review only - http://bmjopen.bmj.com/site/about/guidelines.xhtml

Descriptive clinical characteristics, co-morbidities and survival

A higher proportion of more preterm babies received PNC (<27 weeks 26% (3525/13,527) vs <32 weeks 8% (4713/58,592)). Of those who received PNC, the median (IQR) days to extubation after PNC administration were similar around 5 (2-13) among <32 and <27 gestational week groups (Table 2). Babies who received PNC had a higher incidence of comorbidities and a longer length of stay compared to those who did not (Table 2).

For babies born <27 week, there was higher survival for babies who received vs did not receive PNC (87% vs 70%). However, 80% of deaths among those who did not receive PNC occurred within the first 2 weeks after birth (median (IQR) postnatal day of death 3(1-12) days) compared to 5% of deaths occurring within first 2 weeks among those who received PNC (postnatal day of death 48 (29-96) days).

Table 2 Clinical characteristics, survival and co-morbidities of babies who did and did not receive postnatal steroids presented by <32 and <27 weeks gestation groups

	No postnatal steroids given for BPD, born <32 weeks GA	*Postnatal steroids for BPD, born <32 weeks GA*	*No postnatal steroids given for BPD, born <27 weeks GA*	*Postnatal steroids for BPD, born <27 weeks*
	n= 62019		*n=13527*	
n (%)	57306 (92%)	4713 (8%)	10002 (74%)	3525 (26%)
GA at birth (weeks) Median (IQR)	29.0 (27.2, 31.4)	25.4 (24.4, 27.0)	25.3 (24.1, 26.5)	24.9 (24.1, 25.7)
Birthweight (g) Median (IQR)	1230 (946, 1510)	720 (620, 860)	760 (648, 880)	690 (607, 795)
Male sex	31117 (54%)	2786 (59%)	5301 (53%)	2030 (58%)

For peer review only - http://bmjopen.bmj.com/site/about/guidelines.xhtml

Total length of stay (days) Median (IQR)	47 (33,70)	113 (92,137)	88 (60,108)	118 (99,141)
Total days mechanical ventilation (days) Median (IQR)	2 (0, 5)	33 (20, 48)	17 (4, 24)	37 (25, 51)
Days to extubation after PNC administered Median (IQR)	NA	*5 (2,13)	NA	**5 (2,12)
Extubation<br>1.Extubated and survived<br>2.Extubated and died<br>3.Not extubated after intubation<br>4.Not intubated*	NA	1.2768 (59%)<br>2.546 (12%)<br>3.1333 (28%)<br>4. 66 (1%)	NA	1.2160 (61%)<br>2.420 (12%)<br>3.927 (26%)<br>4.18 (1%)
Survival to discharge	52498 (91.6%)	4098 (87.0%)	6981 (69.8%)	3052 (86.6%)
BPD status	15473 (27%)	4289 (91.0%)	7101 (71.0%)	3285 (93.2%)
Treated Retinopathy of prematurity	1247 (2.2%)	1302 (27.6%)	880 (8.8%)	1166 (33.0%)
Severe NEC	1143 (2.0%)	693 (14.7%)	643 (6.4%)	296 (8.4%)
GI perforation	877 (1.5%)	290 (6.2%)	536 (5.4%)	253 (7.2%)
Brain injury on imaging	2212 (3.9%)	443 (9.4%)	1105 (11.1%)	377 (10.7%)

*3314 babies were extubated following PNC
**2580 babies <27 weeks were extubated following PNC
PNC postnatal corticosteroids

For peer review only

Discussion

We show that in England and Wales, PNC use in preterm babies to prevent or treat BPD, increased between 2012-2019 with about one in four babies born less than 27 weeks gestation receiving a steroid, most commonly dexamethasone, at a postnatal age of around three weeks. Late hydrocortisone and inhaled budesonide were the next most used, with early hydrocortisone administered very rarely. Our findings reflect current guidance from the UK National Institute for Health and Care Excellence (NICE), which recommends limiting the use of dexamethasone to babies after the age of 8 days to assist in weaning from ventilatory support, and not as ‘prophylaxis’ in younger infants. No recommendation is provided for hydrocortisone or nebulised budesonide given limited evidence (8).

The strengths of our study include the large, population representative population of all preterm babies born in England and Wales over an eight-year period. This allowed time trends to be evaluated and for findings to be widely applicable to UK neonatal units. The main limitation of this study is the lack of data on the indication for PNC. The pragmatic

For peer review only - http://bmjopen.bmj.com/site/about/guidelines.xhtml

definition of dexamethasone ≥ 5 consecutive days is likely to capture dexamethasone use for BPD versus upper airway pathology. However the definition for *late* hydrocortisone (started after postnatal day 2 and given for >2 consecutive and >7 days total duration), may still misclassify babies given hydrocortisone for blood pressure support or adrenal insufficiency and may therefore have resulted in an overestimation of the number of babies exposed to PNC for BPD treatment or prevention. As the NNRD contains routine clinical data, we also cannot exclude the possibility of missing data leading to an underestimation of PNC use. We were unable to report cumulative doses of dexamethasone as this is not available in the NNRD.

Studies in other countries have reported similar use of PNC. The 2011 EPICE study found that 13.9% of babies born 24-29 weeks received PNC for BPD, but rates varied from 3.1% (Portugal) to 49.4% (Germany) (26). In a randomised controlled trial (RCT) of erythropoietin versus placebo involving 30 units in the US, 38% of babies born 24 to 27+6 weeks who survived to discharge were treated with PNC (27). The median (IQR) postnatal age for dexamethasone initiation was very similar to our study 29 (20-44) days with a similar duration 10 (5-15) days but use of prednisolone or methylprednisolone was earlier. Studies in other countries have also reported the use of inhaled corticosteroids for BPD (25% and 80% in the United States (28) and Japan (29) respectively).

Given the observational nature of this study, it is unsurprising that babies who received PNC were more preterm and sicker. We did not apply causal inference methods such as propensity matching to examine the associations between PNC and outcomes because achieving good matching was unlikely given the marked differences in characteristics between babies who did and did not receive PNC. Survival was higher among babies born <27 weeks who received PNC, likely due to survival bias. This occurs when patients are expected to survive until the exposure. Patients who die before they have had the opportunity to be exposed will be "unexposed" by definition, and this introduces an artificial survival advantage associated with the exposed subjects regardless of treatment effectiveness (30). The majority of deaths occurred in the first 2 weeks following birth, prior to the median age of dexamethasone initiation (19 days).

Postnatal corticosteroids are one of the most investigated medications in neonatology with over 80 RCT enrolling over 9,000 babies. Despite multiple trials and systematic reviews, (10, 11, 31, 32), there remains uncertainty about the appropriate formulation, timing, dosage, and duration of PNC. Further RCT on dosing and timing of dexamethasone are unlikely to be feasible given that pilot studies, such as the MINIDEX study did not demonstrate feasibility (33, 34), and the DART study (35, 36) failed to recruit target numbers and was abandoned. The recently published results of a RCT involving 800 babies born less than 30 weeks

For peer review only - http://bmjopen.bmj.com/site/about/guidelines.xhtml

showed that a 10 day course of hydrocortisone at 14-28 days in babies who have been intubated for at least 7 days, did not result in substantially higher survival without moderate or severe BPD compared to placebo. They also showed that survival without moderate or severe neurodevelopmental impairment did not differ substantially between the two groups (37). Given the lack of benefit, and potential harms such as gastrointestinal perforation, courses of hydrocortisone at 2-3 weeks old to treat BPD should probably be avoided.

We found increasing use of inhaled budesonide in our study at around 6 weeks of age. A major rationale for inhaled corticosteroids is to avoid some of the complications of systemic corticosteroids by concentrating the drug in the lung. However, given the lack of evidence for early or later inhaled corticosteroids, they are not recommended for routine clinical practice (38, 39). Of note, despite widespread concern regarding long-term effects, few studies have been powered to examine neurodevelopmental and cognitive outcomes. Although less meaningful to parents, and a poor predictor of future functional impairment, most PNC studies have used BPD (oxygen dependency at 36 weeks corrected) as a primary outcome. We have shown that around 400 babies each year are exposed to postnatal dexamethasone and the majority survive to discharge. We suggest that long-term neurodevelopmental surveillance, should be considered mandatory in these infants, and an essential component of good clinical practice. Linking together routine health and educational datasets could be a potential more affordable and practical solution.

Conclusion

In England and Wales, around 1 in 12 babies born less than 32 weeks and 1 in 4 born less than 27 weeks receive postnatal corticosteroids to prevent or treat bronchopulmonary dysplasia. Given the lack of convincing evidence of efficacy, challenges of recruiting to and length of time taken to conduct RCT, our data highlight the need to monitor long-term outcomes in children who receive postnatal corticosteroids.

Ethical approval statement

The study is covered by Research Ethics Committee Approval reference 16/LO/1093.

Contributorship statement

CB, CG and SU conceived this project. SY carried out the analyses. The first draft of the manuscript was written by SY and revised by CB, CG, SU and NM. All authors have reviewed and approved the final manuscript.

For peer review only - http://bmjopen.bmj.com/site/about/guidelines.xhtml

Competing interests

CG is funded by the United Kingdom Medical Research Council (MRC) through a Transition Support Award. He has received support from Chiesi Pharmaceuticals to attend an educational conference; in the past 5 years he has been investigator on received research grants from Medical Research Council, National Institute of Health Research, Canadian Institute of Health Research, Department of Health in England, Mason Medical Research Foundation, Westminster Medical School Research Trust and Chiesi Pharmaceuticals. CG chairs the National Institute for Health and Care Research (NIHR) Research for Patient Benefit London Regional Panel. CB is funded by the UK NIHR through an Advanced Fellowship Award. CB is deputy chair for the NIHR Prioritisation committee for Hospital-based care.

Funding statement

The funding for creating and maintaining the NNRD is from unrestricted funding awarded to NM. This includes costs involved in data transfer, storage, cleaning, merging, administration and regulatory approvals. SY undertook this project as part of the Imperial College Medical School Bachelor of Sciences programme. CB is funded by the United National Institute for Health Research through an Advanced Fellowship (NIHR 300617). CG is funded by the Medical Research Council (MRC) award.

Data sharing statement

The data that support the findings of this study are available from the corresponding author CB, upon reasonable request.

References

1. Siffel C, Kistler KD, Lewis JFM, Sarda SP. Global incidence of bronchopulmonary dysplasia among extremely preterm infants: a systematic literature review. Journal of Maternal-Fetal & Neonatal Medicine. 2021;34(11):1721-31.
2. Bancalari E, Jain D. Bronchopulmonary Dysplasia: 50 Years after the Original Description. Neonatology. 2019;115(4):384-91.
3. Anderson PJ, Doyle LW. Neurodevelopmental outcome of bronchopulmonary dysplasia. Semin Perinatol. 2006;30(4):227-32.
4. Doyle LW, Carse E, Adams AM, Ranganathan S, Opie G, Cheong JLY. Ventilation in Extremely Preterm Infants and Respiratory Function at 8 Years. N Engl J Med. 2017;377(4):329-37.
5. Jobe AH, Bancalari E. Bronchopulmonary dysplasia: Am J Respir Crit Care Med. 2001 Jun;163(7):1723-9. doi: 10.1164/ajrccm.163.7.2011060.
6. Barrington KJ. The adverse neuro-developmental effects of postnatal steroids in the preterm infant: a systematic review of RCTs. BMC Pediatr. 2001;1:1.
7. Doyle LW, Anderson PJ. Long-term outcomes of bronchopulmonary dysplasia. Semin Fetal Neonatal Med. 2009;14(6):391-5.

For peer review only - http://bmjopen.bmj.com/site/about/guidelines.xhtml

8. National Institute for Health and Care Excellence. Respiratory support for preterm babies 2019 [Available from: https://www.nice.org.uk/guidance/ng124/chapter/Recommendations#respiratory-support-for-preterm-babies.
9. Baud O, Zupan V, Lacaze-Masmonteil T, Dehan M. Neurological adverse effects of early postnatal dexamethasone in very preterm infants. Archives of disease in childhood Fetal and neonatal edition. 1999;80(2):F159-F.
10. Doyle LW, Cheong JL, Hay S, Manley BJ, Halliday HL. Early (< 7 days) systemic postnatal corticosteroids for prevention of bronchopulmonary dysplasia in preterm infants. Cochrane Database of Systematic Reviews. 2021(10).
11. Doyle LW, Cheong JL, Hay S, Manley BJ, Halliday HL. Late (≥ 7 days) systemic postnatal corticosteroids for prevention of bronchopulmonary dysplasia in preterm infants. Cochrane Database of Systematic Reviews. 2021(11).
12. Ramaswamy VV, Bandyopadhyay T, Nanda D, Bandiya P, Ahmed J, Garg A, et al. Assessment of Postnatal Corticosteroids for the Prevention of Bronchopulmonary Dysplasia in Preterm Neonates: A Systematic Review and Network Meta-analysis. Jama, Pediatr. 2021;175(6):e206826-e.
13. Gortner L, Misselwitz B, Milligan D, Zeitlin J, Kollée L, Boerch K, et al. Rates of bronchopulmonary dysplasia in very preterm neonates in Europe: results from the MOSAIC cohort. Neonatology. 2011;99(2):112-7.
14. Nuytten A, Behal H, Duhamel A, Jarreau P-H, Mazela J, Milligan D, et al. Evidence-Based Neonatal Unit Practices and Determinants of Postnatal Corticosteroid-Use in Preterm Births below 30 Weeks GA in Europe. A Population-Based Cohort Study. PLOS ONE. 2017;12(1):e0170234.
15. Watterberg KL. Policy statement--postnatal corticosteroids to prevent or treat bronchopulmonary dysplasia. Pediatrics. 2010;126(4):800-8.
16. Sweet DG, Carnielli V, Greisen G, Hallman M, Ozek E, Te Pas A, et al. European Consensus Guidelines on the Management of Respiratory Distress Syndrome - 2019 Update. Neonatology. 2019;115(4):432-50.
17. Lemyre B, Dunn M, Thebaud B. Postnatal corticosteroids to prevent or treat bronchopulmonary dysplasia in preterm infants. Paediatr Child Health. 2020;25(5):322-31.
18. Battersby C, Statnikov Y, Santhakumaran S, Gray D, Modi N, Costeloe K, et al. The United Kingdom National Neonatal Research Database: A validation study. PLoS One. 2018;13(8):e0201815.
19. Neonatal Data Analysis Unit. National Neonatal Research Database (NNRD) [Available from: www.imperial.ac.uk/neonatal-data-analysis-unit/neonatal-data-analysis-unit.
20. Gale C, Statnikov Y, Jawad S, Uthaya SN, Modi N. Neonatal brain injuries in England: population-based incidence derived from routinely recorded clinical data held in the National Neonatal Research Database. Archives of Disease in Childhood - Fetal and Neonatal Edition. 2018;103(4):F301-F6.
21. Webbe JWH, Duffy JMN, Afonso E, Al-Muzaffar I, Brunton G, Greenough A, et al. Core outcomes in neonatology: development of a core outcome set for neonatal research. Arch Dis Child Fetal Neonatal Ed. 2020;105(4):425-31.
22. Baud O, Maury L, Lebail F, Ramful D, El Moussawi F, Nicaise C, et al. Effect of early low-dose hydrocortisone on survival without bronchopulmonary dysplasia in extremely preterm infants (PREMILOC): a double-blind, placebo-controlled, multicentre, randomised trial. The Lancet. 2016;387(10030):1827-36.
23. Royal College of Paediatrics and Child Health. National Neonatal Audit Programme: A guide to the 2020 audit measures 2020 [Available from: https://www.rcpch.ac.uk/sites/default/files/2019-11/nnap_2020_audit_measures_guide_v1.0_191119_0.pdf.
24. Battersby C, Longford N, Mandalia S, Costeloe K, Modi N. Incidence and enteral feed antecedents of severe neonatal necrotising enterocolitis across neonatal networks in England, 2012-3: a whole-population surveillance study. The Lancet Gastroenterology & Hepatology. 2017;2(1):43-51.

For peer review only - http://bmjopen.bmj.com/site/about/guidelines.xhtml

25. British Association of Perinatal Medicine. Perinatal Management of Extreme Preterm Birth Before 27 weeks of Gestation (2019), A BAPM Framework for Practice 2019 [Available from: https://www.bapm.org/resources/80-perinatal-management-of-extreme-preterm-birth-before-27-weeks-of-gestation-2019.
26. Nuytten A, Behal H, Duhamel A, Jarreau P-H, Mazela J, Milligan D, et al. Evidence-Based Neonatal Unit Practices and Determinants of Postnatal Corticosteroid-Use in Preterm Births below 30 Weeks GA in Europe. A Population-Based Cohort Study. PloS one. 2017;12(1):e0170234-e.
27. Puia-Dumitrescu M, Wood TR, Comstock BA, Law JB, German K, Perez KM, et al. Dexamethasone, Prednisolone, and Methylprednisolone Use and 2-Year Neurodevelopmental Outcomes in Extremely Preterm Infants. JAMA Network Open. 2022;5(3):e221947-e.
28. Slaughter JL, Stenger MR, Reagan PB, Jadcherla SR. Utilization of inhaled corticosteroids for infants with bronchopulmonary dysplasia. PLoS One. 2014;9(9):e106838.
29. Ogawa R, Mori R, Sako M, Kageyama M, Tamura M, Namba F. Drug treatment for bronchopulmonary dysplasia in Japan: questionnaire survey. Pediatr Int. 2015;57(1):189-92.
30. Zhou Z, Rahme E, Abrahamowicz M, Pilote L. Survival bias associated with time-to-treatment initiation in drug effectiveness evaluation: a comparison of methods. Am J Epidemiol. 2005;162(10):1016-23.
31. Shah VS, Ohlsson A, Halliday HL, Dunn M. Early administration of inhaled corticosteroids for preventing chronic lung disease in very low birth weight preterm neonates. Cochrane Database Syst Rev. 2017;1(1):Cd001969.
32. Onland W, Offringa M, van Kaam A. Late (≥ 7 days) inhalation corticosteroids to reduce bronchopulmonary dysplasia in preterm infants. Cochrane Database of Systematic Reviews. 2017(8).
33. Yates H, Chiocchia V, Linsell L, Orsi N, Juszczak E, Johnson K, et al. Very low-dose dexamethasone to facilitate extubation of preterm babies at risk of bronchopulmonary dysplasia: the MINIDEX feasibility RCT. 2019;6:8.
34. Yates H, Chiocchia V, Linsell L, Orsi N, Juszczak E, Johnson K, et al. Efficacy and Mechanism Evaluation. Very low-dose dexamethasone to facilitate extubation of preterm babies at risk of bronchopulmonary dysplasia: the MINIDEX feasibility RCT. Southampton (UK)2019.
35. Doyle LW, Davis PG, Morley CJ, McPhee A, Carlin JB. Low-dose dexamethasone facilitates extubation among chronically ventilator-dependent infants: a multicenter, international, randomized, controlled trial. Pediatrics. 2006;117(1):75-83.
36. Doyle LW, Davis PG, Morley CJ, McPhee A, Carlin JB. Outcome at 2 years of age of infants from the DART study: a multicenter, international, randomized, controlled trial of low-dose dexamethasone. Pediatrics. 2007;119(4):716-21.
37. Watterberg KL, Walsh MC, Li L, Chawla S, D'Angio CT, Goldberg RN, et al. Hydrocortisone to Improve Survival without Bronchopulmonary Dysplasia. New England Journal of Medicine. 2022;386(12):1121-31.
38. Bassler D, Plavka R, Shinwell ES, Hallman M, Jarreau PH, Carnielli V, et al. Early Inhaled Budesonide for the Prevention of Bronchopulmonary Dysplasia. New England Journal of Medicine. 2015;373(16):1497-506.
39. Bassler D, Shinwell ES, Hallman M, Jarreau PH, Plavka R, Carnielli V, et al. Long-Term Effects of Inhaled Budesonide for Bronchopulmonary Dysplasia. New England Journal of Medicine. 2018;378(2):148-57.

DECLARATIONS

Consent for publication

Not applicable

For peer review only - http://bmjopen.bmj.com/site/about/guidelines.xhtml

Availability of data and material

Applications to use the data used within this project should be made to the Neonatal Data Analysis Unit, Imperial College London

Acknowledgements

We wish to acknowledge all neonatal units in the UK Neonatal collaborative who contribute data to the NNRD*

What is known about the subject

Bronchopulmonary dysplasia (BPD) is the most common complication of preterm birth.

The use, choice and timing of postnatal corticosteroid remains controversial due to the increased risk of neurodevelopmental impairment associated with high-dose steroids.

What this study adds

Between 2012-2019 in England and Wales, around 8% of babies born <32 weeks received postnatal corticosteroid; use was highest in the most preterm with one in four of babies born <27 gestational weeks given PNC.

The most commonly used PNC was dexamethasone (5%).

Use of dexamethasone, hydrocortisone, and inhaled budesonide have all increased over time, while the use of prednisolone and methylprednisolone remained stable or declined.

Figure legends

Figure 1. Flow chart of cohort who received postnatal steroids*

*babies can have more than one postnatal corticosteroid type

Figure 2. Graph showing the use of postnatal corticosteroids over time

Figure 3. A boxplot showing the median, IQR and range of first day postnatal corticosteroid administration for BPD *(grouped by postnatal corticosteroid type)* in babies who were born at <32 weeks gestation between 2012-2019 and were admitted to neonatal units in England and Wales

For peer review only

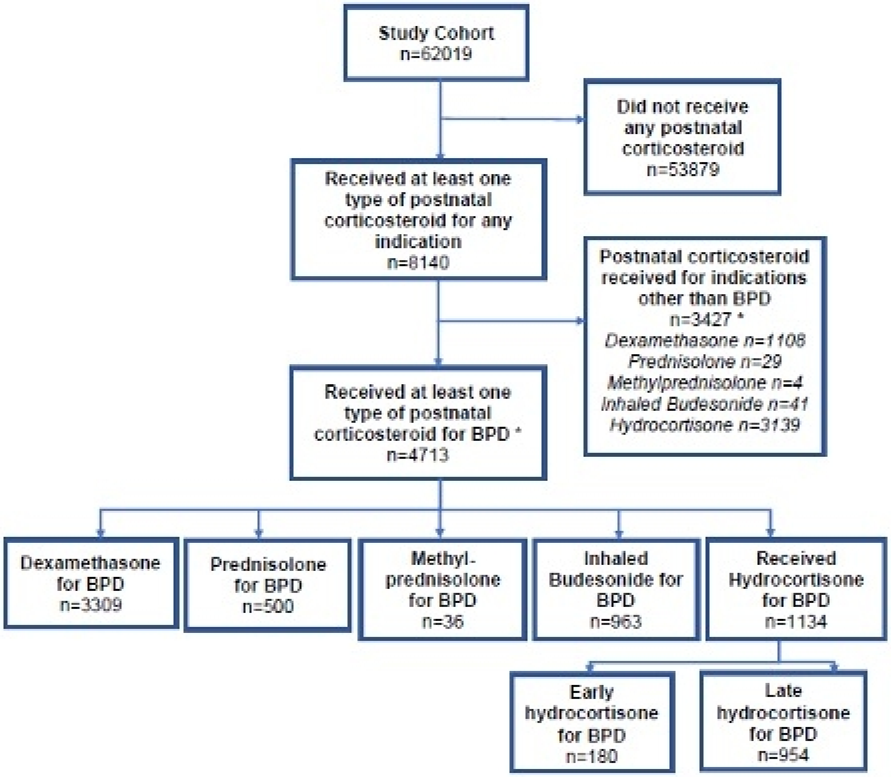

Figure 1. Flow chart of cohort who received postnatal steroids
*babies can have more than one postnatal corticosteroid type

157x147mm (72 x 72 DPI)

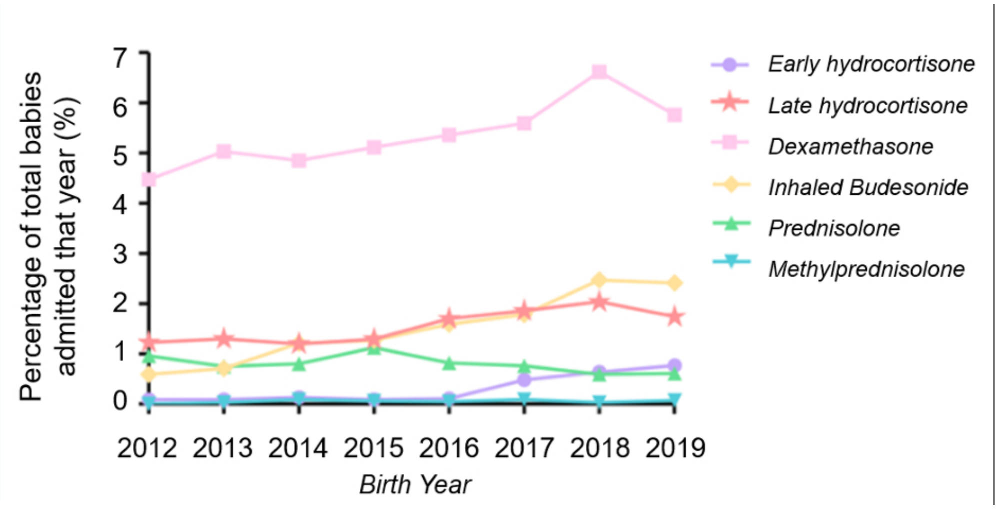

Figure 2. Graph showing the use of postnatal corticosteroids over time

159x79mm (150 x 150 DPI)

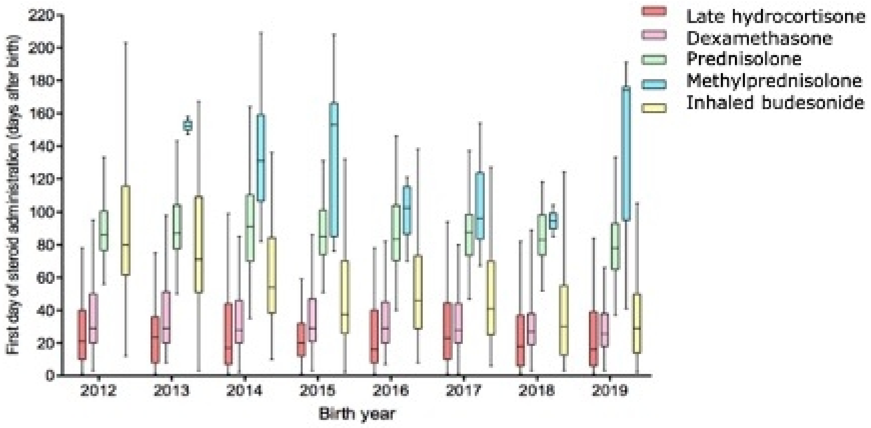

Figure 3. Boxplot showing the median, IQR and range of first day postnatal corticosteroid administration for BPD (grouped by postnatal corticosteroid type) in babies who were born at <32 weeks gestation between 2012-2019 and were admitted to neonatal units in England and Wales

159x83mm (72 x 72 DPI)

Supplementary file S1

Code	Diagnosis
15817	Neonatal intestinal perforation
15819	Neonatal ileum perforation
15821	Neonatal terminal ileum perforation
11010244	Intestinal perforation - not NEC
11217	Closure of jejunal perforation
11222	Closure of small intestine/ileal perforation

For peer review only

The RECORD statement – checklist of items, extended from the STROBE statement, that should be reported in observational studies using routinely collected health data.

	Item No.	STROBE items	Location in manuscript where items are reported	RECORD items	Location in manuscript where items are reported
**Title and abstract**					
	1	(a) Indicate the study's design with a commonly used term in the title or the abstract (b) Provide in the abstract an informative and balanced summary of what was done and what was found		RECORD 1.1: The type of data used should be specified in the title or abstract. When possible, the name of the databases used should be included.<br><br>RECORD 1.2: If applicable, the geographic region and timeframe within which the study took place should be reported in the title or abstract.<br><br>RECORD 1.3: If linkage between databases was conducted for the study, this should be clearly stated in the title or abstract.	page 2
**Introduction**					
Background rationale	2	Explain the scientific background and rationale for the investigation being reported			Page 4
Objectives	3	State specific objectives, including any prespecified hypotheses			Page 5
**Methods**					
Study Design	4	Present key elements of study design early in the paper			Page 5 and 6
Setting	5	Describe the setting, locations, and relevant dates, including periods of recruitment, exposure, follow-up, and data collection			Page 5

For peer review only

Participants	6	*(a) Cohort study* - Give the eligibility criteria, and the sources and methods of selection of participants. Describe methods of follow-up<br>*Case-control study* - Give the eligibility criteria, and the sources and methods of case ascertainment and control selection. Give the rationale for the choice of cases and controls<br>*Cross-sectional study* - Give the eligibility criteria, and the sources and methods of selection of participants<br><br>*(b) Cohort study* - For matched studies, give matching criteria and number of exposed and unexposed<br>*Case-control study* - For matched studies, give matching criteria and the number of controls per case		RECORD 6.1: The methods of study population selection (such as codes or algorithms used to identify subjects) should be listed in detail. If this is not possible, an explanation should be provided.<br><br>RECORD 6.2: Any validation studies of the codes or algorithms used to select the population should be referenced. If validation was conducted for this study and not published elsewhere, detailed methods and results should be provided.<br><br>RECORD 6.3: If the study involved linkage of databases, consider use of a flow diagram or other graphical display to demonstrate the data linkage process, including the number of individuals with linked data at each stage.	Page 5
Variables	7	Clearly define all outcomes, exposures, predictors, potential confounders, and effect modifiers. Give diagnostic criteria, if applicable.		RECORD 7.1: A complete list of codes and algorithms used to classify exposures, outcomes, confounders, and effect modifiers should be provided. If these cannot be reported, an explanation should be provided.	Page 6 and supplementary
Data sources/ measurement	8	For each variable of interest, give sources of data and details of methods of assessment (measurement).<br>Describe comparability of assessment methods if there is more than one group			Page 5

For peer review only

Bias	9	Describe any efforts to address potential sources of bias			Page 5
Study size	10	Explain how the study size was arrived at			Page 5
Quantitative variables	11	Explain how quantitative variables were handled in the analyses. If applicable, describe which groupings were chosen, and why			Page 6
Statistical methods	12	(a) Describe all statistical methods, including those used to control for confounding<br>(b) Describe any methods used to examine subgroups and interactions<br>(c) Explain how missing data were addressed<br>(d) *Cohort study* - If applicable, explain how loss to follow-up was addressed<br>*Case-control study* - If applicable, explain how matching of cases and controls was addressed<br>*Cross-sectional study* - If applicable, describe analytical methods taking account of sampling strategy<br>(e) Describe any sensitivity analyses			Page 6
Data access and cleaning methods		..		RECORD 12.1: Authors should describe the extent to which the investigators had access to the database population used to create the study population.	Page 5

For peer review only

				RECORD 12.2: Authors should provide information on the data cleaning methods used in the study.	
Linkage		..		RECORD 12.3: State whether the study included person-level, institutional-level, or other data linkage across two or more databases. The methods of linkage and methods of linkage quality evaluation should be provided.	N/A
**Results**					
Participants	13	(a) Report the numbers of individuals at each stage of the study (*e.g.*, numbers potentially eligible, examined for eligibility, confirmed eligible, included in the study, completing follow-up, and analysed)<br>(b) Give reasons for non-participation at each stage.<br>(c) Consider use of a flow diagram		RECORD 13.1: Describe in detail the selection of the persons included in the study (*i.e.,* study population selection) including filtering based on data quality, data availability and linkage. The selection of included persons can be described in the text and/or by means of the study flow diagram.	Page 6
Descriptive data	14	(a) Give characteristics of study participants (*e.g.*, demographic, clinical, social) and information on exposures and potential confounders<br>(b) Indicate the number of participants with missing data for each variable of interest<br>(c) *Cohort study* - summarise follow-up time (*e.g.*, average and total amount)			Page 7
Outcome data	15	*Cohort study* - Report numbers of outcome events or summary measures over time<br>*Case-control study* - Report numbers in each exposure			Page 8-10

		category, or summary measures of exposure<br>*Cross-sectional study* - Report numbers of outcome events or summary measures			
Main results	16	(a) Give unadjusted estimates and, if applicable, confounder-adjusted estimates and their precision (e.g., 95% confidence interval). Make clear which confounders were adjusted for and why they were included<br>(b) Report category boundaries when continuous variables were categorized<br>(c) If relevant, consider translating estimates of relative risk into absolute risk for a meaningful time period			Page 8-10
Other analyses	17	Report other analyses done—e.g., analyses of subgroups and interactions, and sensitivity analyses			
**Discussion**					
Key results	18	Summarise key results with reference to study objectives			Page 11
Limitations	19	Discuss limitations of the study, taking into account sources of potential bias or imprecision. Discuss both direction and magnitude of any potential bias		RECORD 19.1: Discuss the implications of using data that were not created or collected to answer the specific research question(s). Include discussion of misclassification bias, unmeasured confounding, missing data, and changing eligibility over time, as they pertain to the study being reported.	Page 11
Interpretation	20	Give a cautious overall interpretation of results considering objectives,			Page 11 and 12

For peer review only

		limitations, multiplicity of analyses, results from similar studies, and other relevant evidence			
Generalisability	21	Discuss the generalisability (external validity) of the study results			Page 11
**Other Information**					
Funding	22	Give the source of funding and the role of the funders for the present study and, if applicable, for the original study on which the present article is based			Page 3
Accessibility of protocol, raw data, and programming code		..		RECORD 22.1: Authors should provide information on how to access any supplemental information such as the study protocol, raw data, or programming code.	Supplementary

*Reference: Benchimol EI, Smeeth L, Guttmann A, Harron K, Moher D, Petersen I, Sørensen HT, von Elm E, Langan SM, the RECORD Working Committee. The REporting of studies Conducted using Observational Routinely-collected health Data (RECORD) Statement. *PLoS Medicine* 2015; in press.

*Checklist is protected under Creative Commons Attribution (CC BY) license.

For peer review only

BMJ Open

Postnatal corticosteroid use for prevention or treatment of Bronchopulmonary Dysplasia in England and Wales 2012-2019: a retrospective population cohort study

Journal:	*BMJ Open*
Manuscript ID	bmjopen-2022-063835.R1
Article Type:	Original research
Date Submitted by the Author:	13-Sep-2022
Complete List of Authors:	Yao, Sijia; Imperial College London, Neonatal Medicine<br>Uthaya, Sabita; Imperial College London, Neonatal Medicine, School of Public Health<br>Gale, Chris; Imperial College London, Neonatal Medicine, School of Public Health<br>Modi, Neena; Imperial College London, Neonatal Medicine, School of Public Health<br>Battersby, Cheryl; Imperial College London, Neonatal Medicine, School of Public Health
**Primary Subject Heading**:	Paediatrics
Secondary Subject Heading:	Respiratory medicine
Keywords:	NEONATOLOGY, Neonatal intensive & critical care < INTENSIVE & CRITICAL CARE, RESPIRATORY MEDICINE (see Thoracic Medicine)

SCHOLARONE™
Manuscripts

I, the Submitting Author has the right to grant and does grant on behalf of all authors of the Work (as defined in the below author licence), an exclusive licence and/or a non-exclusive licence for contributions from authors who are: i) UK Crown employees; ii) where BMJ has agreed a CC-BY licence shall apply, and/or iii) in accordance with the terms applicable for US Federal Government officers or employees acting as part of their official duties; on a worldwide, perpetual, irrevocable, royalty-free basis to BMJ Publishing Group Ltd ("BMJ") its licensees and where the relevant Journal is co-owned by BMJ to the co-owners of the Journal, to publish the Work in this journal and any other BMJ products and to exploit all rights, as set out in our licence.

The Submitting Author accepts and understands that any supply made under these terms is made by BMJ to the Submitting Author unless you are acting as an employee on behalf of your employer or a postgraduate student of an affiliated institution which is paying any applicable article publishing charge ("APC") for Open Access articles. Where the Submitting Author wishes to make the Work available on an Open Access basis (and intends to pay the relevant APC), the terms of reuse of such Open Access shall be governed by a Creative Commons licence – details of these licences and which Creative Commons licence will apply to this Work are set out in our licence referred to above.

Other than as permitted in any relevant BMJ Author's Self Archiving Policies, I confirm this Work has not been accepted for publication elsewhere, is not being considered for publication elsewhere and does not duplicate material already published. I confirm all authors consent to publication of this Work and authorise the granting of this licence.

For peer review only

Title:

Postnatal corticosteroid use for prevention or treatment of Bronchopulmonary Dysplasia in England and Wales 2012-2019: a retrospective population cohort study

Authors

S Yao[1], S Uthaya[1,2], C Gale[1,2], N Modi[1,2], C Battersby[1,2] on behalf of the UK Neonatal Collaborative

1. Neonatal Medicine, School of Public Health, Faculty of Medicine, Imperial College London, London, United Kingdom
2. Chelsea and Westminster NHS Foundation Trust

Corresponding author

Cheryl Battersby, PhD

Section of Neonatal Medicine, Chelsea and Westminster campus, Imperial College London. 369 Fulham Road, London, SW10 9NH, United Kingdom.

c.battersby@imperial.ac.uk

Keywords

Neonatal; postnatal corticosteroids; preterm; bronchopulmonary dysplasia

2049/2500

For peer review only

Abstract

Objective: Describe the population of babies who do and do not receive postnatal corticosteroids for prevention or treatment of BPD

Design: Retrospective cohort study using data held in the National Neonatal Research Database

Setting: National Health Service neonatal units in England and Wales

Patients: Babies born less than 32 weeks gestation and admitted to neonatal units from 1st January 2012 to 31st December 2019.

Main outcomes: Proportion of babies given postnatal corticosteroid; type of corticosteroid; age at initiation and duration, trends over time

Secondary outcomes: Survival to discharge, treatment for retinopathy of prematurity, bronchopulmonary dysplasia, brain injury, severe necrotising enterocolitis, gastrointestinal perforation.

Results: 8% (4713/62019) of babies born <32 weeks and 26% (3525/13527) born <27 weeks received postnatal corticosteroids for BPD. Dexamethasone was predominantly used 5.3% (3309/62019), followed by late hydrocortisone 1.5%, inhaled budesonide 1.5%. prednisolone 0.8%, early hydrocortisone 0.3% and methylprednisolone 0.05%. Dexamethasone use increased over time (2012: 4.5 vs. 2019: 5.8%, p=0.04). Median postnatal age of initiation of corticosteroid course was around 3 weeks for *late hydrocortisone*, 4 weeks for dexamethasone, 6 weeks for inhaled budesonide,12 weeks for prednisolone and 16 weeks for methylprednisolone. Babies who received postnatal corticosteroids were born more prematurely, had a higher incidence of comorbidities and a longer length of stay.

Conclusions: In England and Wales, around 1 in 12 babies born less than 32 weeks and 1 in 4 born less than 27 weeks receive postnatal corticosteroids to prevent or treat bronchopulmonary dysplasia. Given the lack of convincing evidence of efficacy, challenges of recruiting to and length of time taken to conduct RCT, our data highlight the need to monitor long-term outcomes in children who received neonatal postnatal corticosteroids.

Strengths and limitations of this study

Data for sixty-two thousand and nineteen preterm babies were included in this study

For peer review only - http://bmjopen.bmj.com/site/about/guidelines.xhtml

For peer review only

Exposure to different types of postnatal corticosteroids, including timing of initiation and duration are described

Data over an eight-year period enabled time trends to be evaluated

Lack of data on the indication for postnatal corticosteroid may lead to overestimation of babies exposed to hydrocortisone for BPD versus for blood pressure or adrenal insufficiency

We were unable to report cumulative doses of dexamethasone as this was not available

Introduction

Bronchopulmonary dysplasia (BPD), defined as needing respiratory support at 36 weeks post-menstrual age, is the most common adverse outcome of very preterm delivery, affecting up to 75% of babies born before 28 weeks of gestation worldwide (1, 2). In the most severely affected, supplemental oxygen at home is needed, pulmonary hypertension may develop and lung-function abnormalities can persist into adulthood. BPD is also associated with delayed brain maturation and diffuse white matter anomalies that are associated with increased risk of neurodevelopmental impairment (3). Despite increased use of less invasive ventilation after birth, there has been no significant decline in BPD rates globally, and nor improvement in lung function in childhood over time (4). The pathogenesis of BPD remains to be fully understood but involves arrest of lung development (alveolar hypoplasia or dysmorphic pulmonary vasculature) and pulmonary inflammation. It is therefore postulated that the anti-inflammatory action of postnatal corticosteroids (PNC) may improve gas exchange and lung mechanics, thus facilitating weaning from invasive mechanical ventilation and improving respiratory outcomes (5). However, the use of PNC remains controversial because of fear of long-term adverse neurodevelopmental effects, hypertension, intestinal perforation and impaired glucose metabolism (6-8). Whilst early use of dexamethasone (<7 days) is associated with increased incidence of cerebral palsy (9), there is no strong evidence from available studies to suggest that later (>7 days) administration of lower dose dexamethasone, or early prophylactic use of low dose hydrocortisone, causes neurodevelopmental impairment (10, 11).

A recent systematic review and network meta-analysis included 62 studies involving 5559 neonates with a mean gestational age of 26 weeks and examined 14 different postnatal corticosteroid regimens. This found that a moderately early-initiated (8-14 days), medium cumulative dose of systemic dexamethasone (2-4mg/kg) was the best regimen for preventing mortality or BPD but acknowledged the weak evidence upon which this recommendation is based (12). Hence, when considering PNC treatment clinicians have to

For peer review only - http://bmjopen.bmj.com/site/about/guidelines.xhtml

balance the adverse consequences of long-term ventilation with the potential for long-term neurodisability.

International studies report varying use of PNC (3%-50%) including both systemic and inhaled PNC for preterm neonates (13, 14). Policy recommendations regarding the use of dexamethasone or other corticosteroids, including hydrocortisone and inhaled corticosteroids differ widely (15-17). We aimed to describe the use of PNC for prevention or treatment of BPD over an eight year period in very preterm babies born 2012-2019 in England and Wales, using the National Neonatal Research Database (NNRD). Objectives: i) to quantify the number and proportion of babies who received PNC, timing and duration ii) to describe the clinical characteristics of babies and unadjusted outcomes for babies who do and do not receive PNC

Methods

We undertook a retrospective and descriptive cohort study using deidentified, routinely recorded neonatal clinical data held in the NNRD. The NNRD is a national resource containing detailed, quality assured, clinical information extracted from the electronic patient records of admissions to NHS neonatal units (18, 19). The study is covered by Research Ethics Committee Approval reference 16/LO/1093.

Study population

We included data held in the NNRD on all babies born less than 32 weeks gestation born and admitted to neonatal care units in England and Wales between 1st January 2012 and 31st December 2019. We excluded babies with missing gestational ages. We extracted variables related to maternal factors (ethnicity, age, antenatal steroids); infant demographics (birth weight, sex, gestational age), clinical characteristics (condition at birth including Apgar score and resuscitation details); postnatal corticosteroid use including methylprednisolone, prednisolone, hydrocortisone, dexamethasone, budesonide; respiratory support, co-morbidities (gastrointestinal perforation, bronchopulmonary dysplasia, retinopathy of prematurity that required treatment, brain injury on imaging) (20, 21) and survival to discharge from neonatal care. Data were extracted using SAS version 13.

Definitions

Postnatal corticosteroids may be used for indications other than prevention or treatment of BPD (upper airway obstruction, blood pressure); however indication for postnatal steroid use

For peer review only - http://bmjopen.bmj.com/site/about/guidelines.xhtml

is not held in the NNRD. We therefore applied the following pragmatic definitions to identify babies we considered to have received postnatal steroids for BPD prevention or treatment:

Dexamethasone: Dexamethasone for ≥ 5 consecutive days (5 days was chosen to avoid the inclusion of babies given a shorter course for upper airway obstruction)
Early hydrocortisone: hydrocortisone started on postnatal day 1 or 2 and given for >7 consecutive days consistent with the PREMILOC trial (22).
Late hydrocortisone: hydrocortisone started after postnatal day 2 and given for >2 consecutive and >7 days total duration (to differentiate this from hydrocortisone given for hypotension)

Prednisolone: prednisolone given for ≥ 3 consecutive days

Methylprednisolone: methylprednisolone given for ≥ 3 consecutive days

Inhaled budesonide: Use of inhaled budesonide for ≥ 1 day

Bronchopulmonary dysplasia was defined as receiving any respiratory or ventilatory support or supplemental oxygen at 36 weeks postmenstrual age (23) , treatment for retinopathy of prematurity (ROP) was defined as cryotherapy, laser therapy or injection of anti-vascular endothelial growth factor therapy for ROP in either or both eyes, severe necrotising enterocolitis was defined as necrotising enterocolitis resulting in surgery or confirmed at surgery (24), brain injury was defined as either left or right grade 3 or higher intraventricular haemorrhage or periventricular leukomalacia (20), gastrointestinal perforation was defined as having a perforation if presence of relevant diagnostic and/or procedural codes (Supplemental file S1).

Data analysis

Simple descriptive statistics were used to describe the study population, clinical characteristics, survival and co-morbidities without adjustment. We report results for the entire cohort of babies born <32 weeks and also a subgroup of the most preterm babies born <27 weeks gestation based on the British Association of Perinatal Medicine (BAPM) extreme preterm criteria (25). Data were described using medians and interquartile ranges for continuous and non-parametric (as determined by a Shapiro-Wilk normality test) data, and proportions for categorical data. Temporal trends were assessed using linear regression. For all the statistical results, a p value <0.05 was considered as statistically significant. Analyses were performed using RStudio, SPSS, and GraphPad Prism 5.

Patient and Public Involvement statement

For peer review only

Patients or the public were not involved in the design, or conduct, or reporting, or dissemination plans of our research

Results

Number and proportion of babies over time who received postnatal corticosteroids for BPD

A total of 62,019 babies born <32 weeks gestation were born and admitted to neonatal units in England and Wales during the study period. 8140 (13%) babies were recorded as having received PNC for any indication and 7.6% (4713/62019) received at least one type of PNC for BPD (Figure 1). The most commonly used PNC was *dexamethasone* 5.3% (3309/62019), followed by *late hydrocortisone* 1.5% (954/62019), *inhaled budesonide* 1.5% (922/62019), *prednisolone* 0.8% (500/62019). *Early hydrocortisone* 0.3% (180/62019) and *methylprednisolone* 0.05% (32/62019) use were rare. 6.3% (3914/62019) received only one type of PNC (*early hydrocortisone* 156, *late hydrocortisone* 620, *dexamethasone* 2461, *prednisolone* 196, *inhaled budesonide* 481). 1.3% (799/62019) received more than one type of PNC.

Between 2012 and 2019, an increasing proportion of very preterm babies received PNC (p=0.005) (Figure 2). There was an overall increase use over time for *dexamethasone*, *inhaled budesonide* and *late hydrocortisone* even though there was a small decrease between 2018 and 2019; *dexamethasone* (2012: 4.5% vs. 2018: 6.6% vs. 2019: 5.8%, 2.0% increase per year, p=0.04); *inhaled budesonide* (2012: 0.6% vs.2018: 2.5% vs. 2019: 2.4%, 19.3% increase per year, p<0.001) (922 babies); *late hydrocortisone* (2012: 1.2% vs. 2018: 2.0% vs. 2019: 1.7%, 4.1% increase per year, p =0.02). The number for *early hydrocortisone* was very small (180) and its use was first seen in 2017 and has increased over time (2012: 0.1% vs. 2019: 0.8%, 3.7% increase per year, p=0.05). Use of *prednisolone* has decreased (2012: 1.0% vs. 2019: 0.6%, 7.8% decrease per year, p<0.09) (500 babies) and *methylprednisolone* remained similar over time (2012: 0.0% vs. 2019: 0.1%).

Clinical characteristics of babies overall and by type of postnatal corticosteroid for BPD

The descriptive and clinical characteristics of babies by type of PNC are presented in Table 1. The median (IQR) gestational age for babies who received PNC was 25 (24-27) weeks;

For peer review only - http://bmjopen.bmj.com/site/about/guidelines.xhtml

71% received a complete course of antenatal steroids; 87% were intubated at birth. Overall among the entire cohort less than 32 weeks, the intubation rate at delivery was 51% (31,609/62019) and 92% for less than 27 weeks (12480/13527) (Table 2).

At the time of PNC initiation, 75% and 20% were receiving invasive and non-invasive ventilatory support, respectively, with the exception of inhaled budesonide where 49% were on invasive ventilation; 7% received drugs for resuscitation or cardiac massage at delivery.

Table 1. Clinical characteristics of infants by type of postnatal corticosteroid <32 weeks gestation

	Dexamethasone *(n=3309)*	***Late Hydrocortisone*** *(n=954)*	***Prednisolone*** *(n=500)*	***Methyl-prednisolone*** *(n=32)*	***Inhaled Budesonide*** *(n=922)*	***Early hydrocortisone*** *(N=180*
Gestational age in weeks, median (IQR)	25.1 (24.3, 26.6)	25.4 (24.3, 27.6)	25.4 (24.5, 26.7)	24.9 (24.1, 28.0)	25.7 (24.6, 27.3)	25.0 (24.0, 27.0)
Antenatal corticosteroids						
None	166 (5%)	43 (5%)	26 (5%)	2 (6%)	25 (3%)	3 (2%)
Complete	2306 (70%)	677 (71%)	344 (69%)	24 (75%)	699 (76%)	124 (67%)
Incomplete	574 (17%)	165 (17%)	98 (20%)	5 (16%)	151 (16%)	37 (32%)
Missing	2405 (8%)	667 (7%)	32 (6%)	1 (3%)	46 (5%)	-
Birthweight (g) median (IQR)	710 (620, 840)	700 (600, 854)	710 (615, 84)	755 (597, 869)	758 (640, 910)	757 (645, 916)
Sex Male	1975(60%)	591 (62%)	288 (58%)	22 (69%)	580 (61%)	93 (50%)
Apgar 5 mins						
≤ 5	839 (25%)	231 (24%)	115 (23%)	9 (28%)	218 (24%)	61 (33%)
>5	1960 (59%)	602 (63%)	291 (58%)	20 (63%)	621 (67%)	100 (54%)
Missing	510 (15%)	121 (13%)	94 (19%)	3 (9%)	83 (9%)	23 (13%)
Cardiac massage at delivery	245 (7%)	64 (7%)	31 (6%)	3 (9%)	52 (6%)	18 (10%)
Drugs for resuscitation at delivery	255 (8%)	72 (8%)	50 (10%)	2 (6%)	50 (5%)	20 (11%)

For peer review only

Intubation at delivery	3021 (91%)	838 (88%)	448 (90%)	28 (88%)	698 (76%)	162 (88%)
Postnatal age (days) at initiation of postnatal corticosteroid, median (IQR)	28 (19, 45)	19 (8, 40)	86 (73, 101)	116 (90, 155)	42 (23, 70)	1 (1, 2)
Duration (days), median (IQR)	14 (10, 25)	20 (11, 38)	9 (8, 30)	7 (4, 11)	19 (9,34)	11(10, 14)
Respiratory support at time of steroid initiation<br>*Invasive ventilation*<br>*Non-invasive support*<br>*None of the above*	2799 (85%)<br>445 (13%)<br>64 (2%)	718 (75%)<br>145 (18%)<br>90 (9%)	372 (74%)<br>86 (17%)<br>42 (8%)	26 (81%)<br>4 (13%)<br>2 (6%)	448 (49%)<br>422 (46%)<br>51 (6%)	171 (93%)<br>12 (7%)<br>1 (0.5%)

For peer review only

Timing and duration of postnatal corticosteroids for BPD

Median (IQR) postnatal age at initiation was 19 (8-40) days for *late hydrocortisone*, 28 (19-45) days for *dexamethasone*, 42 (30-70) days for *inhaled budesonide*, 865 (73-101) for *prednisolone* and 116 (90-155) days for methylprednisolone (Table 1). Over time, the postnatal age at initiation for all PNC remained fairly consistent except for *inhaled budesonide* which is being used earlier in recent years (2012 median (IQR) 79 (57,116) vs 2019 30 (14-51)) (Figure 3). The duration of use was shortest for *methylprednisolone* 7 (4-11) days and longest for *late hydrocortisone* 20 (11-38) days (Table 1).

Descriptive clinical characteristics, co-morbidities and survival by gestational age groups

The clinical characteristics, co-morbidities and survival in babies who did and did not receive PNC are presented by gestational subgroups (<32 weeks and <27 weeks) in Table 2. A higher proportion of more preterm babies received PNC (<27 weeks 26% (3525/13,527) vs <32 weeks 8% (4713/58,592)). Of those who received PNC, the median (IQR) days to extubation after PNC administration were similar around 5 (2-13) among <32 and <27 gestational week groups (Table 2). Babies who received PNC had a higher incidence of comorbidities and a longer length of stay compared to those who did not (Table 2).

For babies born <27 week, there was higher survival for babies who received vs did not receive PNC (87% vs 70%). However, 80% of deaths among those who did not receive PNC occurred within the first 2 weeks after birth (median (IQR) postnatal day of death 3(1-12) days) compared to 5% of deaths occurring within first 2 weeks among those who received PNC (postnatal day of death 48 (29-96) days).

Table 2 Clinical characteristics, survival and co-morbidities of babies who did and did not receive postnatal steroids presented by <32 and <27 weeks gestation groups

	No postnatal steroids given for BPD, born <32 weeks GA	*Postnatal steroids for BPD, born <32 weeks GA*	*No postnatal steroids given for BPD, born <27 weeks GA*	*Postnatal steroids for BPD, born <27 weeks*
	n= 62019		*n=13527*	
n (%)	57306 (92%)	4713 (8%)	10002 (74%)	3525 (26%)
GA at birth (weeks) Median (IQR)	29.0 (27.2, 31.4)	25.4 (24.4, 27.0)	25.3 (24.1, 26.5)	24.9 (24.1, 25.7)
Birthweight (g) Median (IQR)	1230 (946, 1510)	720 (620, 860)	760 (648, 880)	690 (607, 795)
Male sex	31117 (54%)	2786 (59%)	5301 (53%)	2030 (58%)
Intubation at birth	27509 (48%)	4100 (87%)	9202 (92.3%)	3278 (93%)
Antenatal corticosteroids				
None	3256 (6%)	220 (5%)	764 (8%)	170 (1%)
Complete	39997 (70%)	3343 (71%)	6754 (68%)	2434 (69%)
Incomplete	10574 (18%)	807 (17%)	2235 (22%)	659 (17%)
Missing	3479 (6%)	343(7%)	249 (2%)	262 (7%)
Apgar 5 mins				
≤ 5	8300 (14%)	1138 (24%)	2699 (27%)	919 (26%)
>5	41445 (72%)	2880 (61%)	6420 (64%)	2091 (59%)
Missing	7561 (13%)	695 (15%)	900 (9%)	515 (15%)

For peer review only - http://bmjopen.bmj.com/site/about/guidelines.xhtml

Cardiac massage at delivery	2441(4%)	315 (7%)	844 (8%)	232 (2%)
Total length of stay (days) Median (IQR)	47 (33,70)	113 (92,137)	88 (60,108)	118 (99,141)
Total days mechanical ventilation (days) Median (IQR)	2 (0, 5)	33 (20, 48)	17 (4, 24)	37 (25, 51)
Days to extubation after PNC administered Median (IQR)	NA	*5 (2,13)	NA	**5 (2,12)
Extubation 1.Extubated and survived 2.Extubated and died 3.Not extubated after intubation 4.Not intubated*	NA	1.2768 (59%) 2.546 (12%) 3.1333 (28%) 4. 66 (1%)	NA	1.2160 (61%) 2.420 (12%) 3.927 (26%) 4.18 (1%)
Survival to discharge	52498 (91.6%)	4098 (87.0%)	6981 (69.8%)	3052 (86.6%)
BPD status	15473 (27%)	4289 (91.0%)	7101 (71.0%)	3285 (93.2%)
Treated Retinopathy of prematurity	1247 (2.2%)	1302 (27.6%)	880 (8.8%)	1166 (33.0%)
Severe NEC	1143 (2.0%)	693 (14.7%)	643 (6.4%)	296 (8.4%)
GI perforation	877 (1.5%)	290 (6.2%)	536 (5.4%)	253 (7.2%)
Brain injury on imaging	2212 (3.9%)	443 (9.4%)	1105 (11.1%)	377 (10.7%)

*3314 babies were extubated following PNC
**2580 babies <27 weeks were extubated following PNC
PNC postnatal corticosteroids

Discussion

We show that in England and Wales, PNC use in preterm babies to prevent or treat BPD, increased between 2012-2019 with about one in four babies born less than 27 weeks gestation receiving a steroid, most commonly dexamethasone, at a postnatal age of around three weeks. Late hydrocortisone and inhaled budesonide were the next most used, with early hydrocortisone administered very rarely. Our findings reflect current guidance from the UK National Institute for Health and Care Excellence (NICE), which recommends limiting the use of dexamethasone to babies after the age of 8 days to assist in weaning from ventilatory support, and not as 'prophylaxis' in younger infants. No recommendation is provided for hydrocortisone or nebulised budesonide given limited evidence (8).

For peer review only

The increase in proportion of infants exposed to PNC is not explained by an increase in extremely preterm infants admitted. The median gestational age in the less than 27 weeks group remained at 25.4 weeks between 2012 and 2019, and the proportion of infants born <27 weeks/<32 weeks (2012: 22% vs 2019: 21%) remained similar over time. The increase is also not explained by survivor bias as the mortality rates before 28 days (the median postnatal age of administering the most commonly used PNC dexamethasone) were similar (2012: 7.8%) vs 2019: (6.8%).

The strengths of our study include the large, population representative population of all preterm babies born in England and Wales over an eight-year period. This allowed time trends to be evaluated and for findings to be widely applicable to UK neonatal units. The main limitation of this study is the lack of data on the indication for PNC. The pragmatic definition of dexamethasone ≥ 5 consecutive days is likely to capture dexamethasone use for BPD versus upper airway pathology. However the definition for *late* hydrocortisone (started after postnatal day 2 and given for >2 consecutive and >7 days total duration), may still misclassify babies given hydrocortisone for blood pressure support or adrenal insufficiency and may therefore have resulted in an overestimation of the number of babies exposed to PNC for BPD treatment or prevention. The possible misclassification of PNC for BPD may also explain the unlikely finding that around 5% of infants were not on any respiratory support (including oxygen) on the day of initiation of PNC. Erroneous or missing daily data on the type of respiratory support could also explain this finding. As the NNRD contains routine clinical data, we also cannot exclude the possibility of missing data leading to an underestimation of PNC use. We were unable to report cumulative doses of dexamethasone as this is not available in the NNRD.

Studies in other countries have reported similar use of PNC. The 2011 EPICE study found that 13.9% of babies born 24-29 weeks received PNC for BPD, but rates varied from 3.1% (Portugal) to 49.4% (Germany) (26). In a randomised controlled trial (RCT) of erythropoietin versus placebo involving 30 units in the US, 38% of babies born 24 to 27+6 weeks who survived to discharge were treated with PNC (27). The median (IQR) postnatal age for dexamethasone initiation was very similar to our study 29 (20-44) days with a similar duration 10 (5-15) days but use of prednisolone or methylprednisolone was earlier. Studies in other countries have also reported the use of inhaled corticosteroids for BPD (25% and 80% in the United States (28) and Japan (29) respectively).

The overall incidence of BPD in the entire study cohort <32 weeks was 31%, and 91% and 27% for babies who did and did not receive PNC, respectively. Given the observational nature of this study, it is unsurprising that babies who received PNC were more preterm and sicker. We did not apply causal inference methods such as propensity matching to examine

For peer review only - http://bmjopen.bmj.com/site/about/guidelines.xhtml

the associations between PNC and outcomes because achieving good matching was unlikely given the marked differences in characteristics between babies who did and did not receive PNC. Survival was higher among babies born <27 weeks who received PNC, likely due to survival bias. This occurs when patients are expected to survive until the exposure. Patients who die before they have had the opportunity to be exposed will be “unexposed” by definition, and this introduces an artificial survival advantage associated with the exposed subjects regardless of treatment effectiveness (30). The majority of deaths occurred in the first 2 weeks following birth, prior to the median age of dexamethasone initiation (19 days).

Postnatal corticosteroids are one of the most investigated medications in neonatology with over 80 RCT enrolling over 9,000 babies. Despite multiple trials and systematic reviews, (10, 11, 31, 32), there remains uncertainty about the appropriate formulation, timing, dosage, and duration of PNC. Further RCT on dosing and timing of dexamethasone are unlikely to be feasible given that pilot studies, such as the MINIDEX study did not demonstrate feasibility (33, 34), and the DART study (35, 36) failed to recruit target numbers and was abandoned. The recently published results of a RCT involving 800 babies born less than 30 weeks showed that a 10 day course of hydrocortisone at 14-28 days in babies who have been intubated for at least 7 days, did not result in substantially higher survival without moderate or severe BPD compared to placebo. They also showed that survival without moderate or severe neurodevelopmental impairment did not differ substantially between the two groups (37). Given the lack of benefit, and potential harms such as gastrointestinal perforation, courses of hydrocortisone at 2-3 weeks old to treat BPD should probably be avoided.

We found increasing use of inhaled budesonide in our study at around 6 weeks of age. A major rationale for inhaled corticosteroids is to avoid some of the complications of systemic corticosteroids by concentrating the drug in the lung. However, given the lack of evidence for early or later inhaled corticosteroids, they are not recommended for routine clinical practice (38, 39). Of note, despite widespread concern regarding long-term effects, few studies have been powered to examine neurodevelopmental and cognitive outcomes. Although less meaningful to parents, and a poor predictor of future functional impairment, most PNC studies have used BPD (oxygen dependency at 36 weeks corrected) as a primary outcome.

We have shown that around 400 babies each year are exposed to postnatal dexamethasone and the majority survive to discharge. We suggest that long-term neurodevelopmental surveillance, should be considered mandatory in these infants, and an essential component of good clinical practice. Linking together routine health and educational datasets could be a potential more affordable and practical solution.

For peer review only - http://bmjopen.bmj.com/site/about/guidelines.xhtml

Conclusion

In England and Wales, around 1 in 12 babies born less than 32 weeks and 1 in 4 born less than 27 weeks receive postnatal corticosteroids to prevent or treat bronchopulmonary dysplasia. Given the lack of convincing evidence of efficacy, challenges of recruiting to and length of time taken to conduct RCT, our data highlight the need to monitor long-term outcomes in children who receive postnatal corticosteroids.

Ethical approval statement

The study is covered by Research Ethics Committee Approval reference 16/LO/1093.

Contributorship statement

CB, CG and SU conceived this project. SY carried out the analyses. The first draft of the manuscript was written by SY and revised by CB, CG, SU and NM. All authors have reviewed and approved the final manuscript.

Competing interests

CG is funded by the United Kingdom Medical Research Council (MRC) through a Transition Support Award. He has received support from Chiesi Pharmaceuticals to attend an educational conference; in the past 5 years he has been investigator on received research grants from Medical Research Council, National Institute of Health Research, Canadian Institute of Health Research, Department of Health in England, Mason Medical Research Foundation, Westminster Medical School Research Trust and Chiesi Pharmaceuticals. CG chairs the National Institute for Health and Care Research (NIHR) Research for Patient Benefit London Regional Panel. CB is funded by the UK NIHR through an Advanced Fellowship Award. CB is deputy chair for the NIHR Prioritisation committee for Hospital-based care.

Funding statement

The funding for creating and maintaining the NNRD is from unrestricted funding awarded to NM. This includes costs involved in data transfer, storage, cleaning, merging, administration and regulatory approvals. SY undertook this project as part of the Imperial College Medical School Bachelor of Sciences programme. CB is funded by the United National Institute for Health Research through an Advanced Fellowship (NIHR 300617). CG is funded by the Medical Research Council (MRC) award.

Data sharing statement

The data that support the findings of this study are available from the corresponding author CB, upon reasonable request.

For peer review only

References

1. Siffel C, Kistler KD, Lewis JFM, Sarda SP. Global incidence of bronchopulmonary dysplasia among extremely preterm infants: a systematic literature review. Journal of Maternal-Fetal & Neonatal Medicine. 2021;34(11):1721-31.
2. Bancalari E, Jain D. Bronchopulmonary Dysplasia: 50 Years after the Original Description. Neonatology. 2019;115(4):384-91.
3. Anderson PJ, Doyle LW. Neurodevelopmental outcome of bronchopulmonary dysplasia. Semin Perinatol. 2006;30(4):227-32.
4. Doyle LW, Carse E, Adams AM, Ranganathan S, Opie G, Cheong JLY. Ventilation in Extremely Preterm Infants and Respiratory Function at 8 Years. N Engl J Med. 2017;377(4):329-37.
5. Jobe AH, Bancalari E. Bronchopulmonary dysplasia: Am J Respir Crit Care Med. 2001 Jun;163(7):1723-9. doi: 10.1164/ajrccm.163.7.2011060.
6. Barrington KJ. The adverse neuro-developmental effects of postnatal steroids in the preterm infant: a systematic review of RCTs. BMC Pediatr. 2001;1:1.
7. Doyle LW, Anderson PJ. Long-term outcomes of bronchopulmonary dysplasia. Semin Fetal Neonatal Med. 2009;14(6):391-5.
8. National Institute for Health and Care Excellence. Respiratory support for preterm babies 2019 [Available from: https://www.nice.org.uk/guidance/ng124/chapter/Recommendations#respiratory-support-for-preterm-babies.
9. Baud O, Zupan V, Lacaze-Masmonteil T, Dehan M. Neurological adverse effects of early postnatal dexamethasone in very preterm infants. Archives of disease in childhood Fetal and neonatal edition. 1999;80(2):F159-F.
10. Doyle LW, Cheong JL, Hay S, Manley BJ, Halliday HL. Early (< 7 days) systemic postnatal corticosteroids for prevention of bronchopulmonary dysplasia in preterm infants. Cochrane Database of Systematic Reviews. 2021(10).
11. Doyle LW, Cheong JL, Hay S, Manley BJ, Halliday HL. Late (≥ 7 days) systemic postnatal corticosteroids for prevention of bronchopulmonary dysplasia in preterm infants. Cochrane Database of Systematic Reviews. 2021(11).
12. Ramaswamy VV, Bandyopadhyay T, Nanda D, Bandiya P, Ahmed J, Garg A, et al. Assessment of Postnatal Corticosteroids for the Prevention of Bronchopulmonary Dysplasia in Preterm Neonates: A Systematic Review and Network Meta-analysis. Jama, Pediatr. 2021;175(6):e206826-e.
13. Gortner L, Misselwitz B, Milligan D, Zeitlin J, Kollée L, Boerch K, et al. Rates of bronchopulmonary dysplasia in very preterm neonates in Europe: results from the MOSAIC cohort. Neonatology. 2011;99(2):112-7.
14. Nuytten A, Behal H, Duhamel A, Jarreau P-H, Mazela J, Milligan D, et al. Evidence-Based Neonatal Unit Practices and Determinants of Postnatal Corticosteroid-Use in Preterm Births below 30 Weeks GA in Europe. A Population-Based Cohort Study. PLOS ONE. 2017;12(1):e0170234.
15. Watterberg KL. Policy statement--postnatal corticosteroids to prevent or treat bronchopulmonary dysplasia. Pediatrics. 2010;126(4):800-8.
16. Sweet DG, Carnielli V, Greisen G, Hallman M, Ozek E, Te Pas A, et al. European Consensus Guidelines on the Management of Respiratory Distress Syndrome - 2019 Update. Neonatology. 2019;115(4):432-50.
17. Lemyre B, Dunn M, Thebaud B. Postnatal corticosteroids to prevent or treat bronchopulmonary dysplasia in preterm infants. Paediatr Child Health. 2020;25(5):322-31.
18. Battersby C, Statnikov Y, Santhakumaran S, Gray D, Modi N, Costeloe K, et al. The United Kingdom National Neonatal Research Database: A validation study. PLoS One. 2018;13(8):e0201815.

For peer review only

19. Neonatal Data Analysis Unit. National Neonatal Research Database (NNRD) [Available from: www.imperial.ac.uk/neonatal-data-analysis-unit/neonatal-data-analysis-unit.
20. Gale C, Statnikov Y, Jawad S, Uthaya SN, Modi N. Neonatal brain injuries in England: population-based incidence derived from routinely recorded clinical data held in the National Neonatal Research Database. Archives of Disease in Childhood - Fetal and Neonatal Edition. 2018;103(4):F301-F6.
21. Webbe JWH, Duffy JMN, Afonso E, Al-Muzaffar I, Brunton G, Greenough A, et al. Core outcomes in neonatology: development of a core outcome set for neonatal research. Arch Dis Child Fetal Neonatal Ed. 2020;105(4):425-31.
22. Baud O, Maury L, Lebail F, Ramful D, El Moussawi F, Nicaise C, et al. Effect of early low-dose hydrocortisone on survival without bronchopulmonary dysplasia in extremely preterm infants (PREMILOC): a double-blind, placebo-controlled, multicentre, randomised trial. The Lancet. 2016;387(10030):1827-36.
23. Royal College of Paediatrics and Child Health. National Neonatal Audit Programme: A guide to the 2020 audit measures 2020 [Available from: https://www.rcpch.ac.uk/sites/default/files/2019-11/nnap_2020_audit_measures_guide_v1.0_191119_0.pdf.
24. Battersby C, Longford N, Mandalia S, Costeloe K, Modi N. Incidence and enteral feed antecedents of severe neonatal necrotising enterocolitis across neonatal networks in England, 2012-3: a whole-population surveillance study. The Lancet Gastroenterology & Hepatology. 2017;2(1):43-51.
25. British Association of Perinatal Medicine. Perinatal Management of Extreme Preterm Birth Before 27 weeks of Gestation (2019), A BAPM Framework for Practice 2019 [Available from: https://www.bapm.org/resources/80-perinatal-management-of-extreme-preterm-birth-before-27-weeks-of-gestation-2019.
26. Nuytten A, Behal H, Duhamel A, Jarreau P-H, Mazela J, Milligan D, et al. Evidence-Based Neonatal Unit Practices and Determinants of Postnatal Corticosteroid-Use in Preterm Births below 30 Weeks GA in Europe. A Population-Based Cohort Study. PloS one. 2017;12(1):e0170234-e.
27. Puia-Dumitrescu M, Wood TR, Comstock BA, Law JB, German K, Perez KM, et al. Dexamethasone, Prednisolone, and Methylprednisolone Use and 2-Year Neurodevelopmental Outcomes in Extremely Preterm Infants. JAMA Network Open. 2022;5(3):e221947-e.
28. Slaughter JL, Stenger MR, Reagan PB, Jadcherla SR. Utilization of inhaled corticosteroids for infants with bronchopulmonary dysplasia. PLoS One. 2014;9(9):e106838.
29. Ogawa R, Mori R, Sako M, Kageyama M, Tamura M, Namba F. Drug treatment for bronchopulmonary dysplasia in Japan: questionnaire survey. Pediatr Int. 2015;57(1):189-92.
30. Zhou Z, Rahme E, Abrahamowicz M, Pilote L. Survival bias associated with time-to-treatment initiation in drug effectiveness evaluation: a comparison of methods. Am J Epidemiol. 2005;162(10):1016-23.
31. Shah VS, Ohlsson A, Halliday HL, Dunn M. Early administration of inhaled corticosteroids for preventing chronic lung disease in very low birth weight preterm neonates. Cochrane Database Syst Rev. 2017;1(1):Cd001969.
32. Onland W, Offringa M, van Kaam A. Late (≥ 7 days) inhalation corticosteroids to reduce bronchopulmonary dysplasia in preterm infants. Cochrane Database of Systematic Reviews. 2017(8).
33. Yates H, Chiocchia V, Linsell L, Orsi N, Juszczak E, Johnson K, et al. Very low-dose dexamethasone to facilitate extubation of preterm babies at risk of bronchopulmonary dysplasia: the MINIDEX feasibility RCT. 2019;6:8.
34. Yates H, Chiocchia V, Linsell L, Orsi N, Juszczak E, Johnson K, et al. Efficacy and Mechanism Evaluation. Very low-dose dexamethasone to facilitate extubation of preterm babies at risk of bronchopulmonary dysplasia: the MINIDEX feasibility RCT. Southampton (UK)2019.
35. Doyle LW, Davis PG, Morley CJ, McPhee A, Carlin JB. Low-dose dexamethasone facilitates extubation among chronically ventilator-dependent infants: a multicenter, international, randomized, controlled trial. Pediatrics. 2006;117(1):75-83.

For peer review only - http://bmjopen.bmj.com/site/about/guidelines.xhtml

36. Doyle LW, Davis PG, Morley CJ, McPhee A, Carlin JB. Outcome at 2 years of age of infants from the DART study: a multicenter, international, randomized, controlled trial of low-dose dexamethasone. Pediatrics. 2007;119(4):716-21.
37. Watterberg KL, Walsh MC, Li L, Chawla S, D'Angio CT, Goldberg RN, et al. Hydrocortisone to Improve Survival without Bronchopulmonary Dysplasia. New England Journal of Medicine. 2022;386(12):1121-31.
38. Bassler D, Plavka R, Shinwell ES, Hallman M, Jarreau PH, Carnielli V, et al. Early Inhaled Budesonide for the Prevention of Bronchopulmonary Dysplasia. New England Journal of Medicine. 2015;373(16):1497-506.
39. Bassler D, Shinwell ES, Hallman M, Jarreau PH, Plavka R, Carnielli V, et al. Long-Term Effects of Inhaled Budesonide for Bronchopulmonary Dysplasia. New England Journal of Medicine. 2018;378(2):148-57.

For peer review only

DECLARATIONS

Consent for publication

Not applicable

Availability of data and material

Applications to use the data used within this project should be made to the Neonatal Data Analysis Unit, Imperial College London

Acknowledgements

We wish to acknowledge all neonatal units who contribute data to the NNRD, known collectively as the UK Neonatal collaborative www.imperial.ac.uk/neonatal-data-analysis-unit/neonatal-data-analysis-unit/list-of-national-neonatal-units/

What is known about the subject

Bronchopulmonary dysplasia (BPD) is the most common complication of preterm birth.

The use, choice and timing of postnatal corticosteroid remains controversial due to the increased risk of neurodevelopmental impairment associated with high-dose steroids.

What this study adds

Between 2012-2019 in England and Wales, around 8% of babies born <32 weeks received postnatal corticosteroid; use was highest in the most preterm with one in four of babies born <27 gestational weeks given PNC.

The most commonly used PNC was dexamethasone (5%).

Use of dexamethasone, hydrocortisone, and inhaled budesonide have all increased over time, while the use of prednisolone and methylprednisolone remained stable or declined.

Figure legends

Figure 1. Flow chart of cohort who received postnatal steroids*

*babies can have more than one postnatal corticosteroid type

Figure 2. Graph showing the use of postnatal corticosteroids over time

Figure 3. A boxplot showing the median, IQR and range of first day postnatal corticosteroid administration for BPD *(grouped by postnatal corticosteroid type)* in babies who were born at <32 weeks gestation between 2012-2019 and were admitted to neonatal units in England and Wales

For peer review only

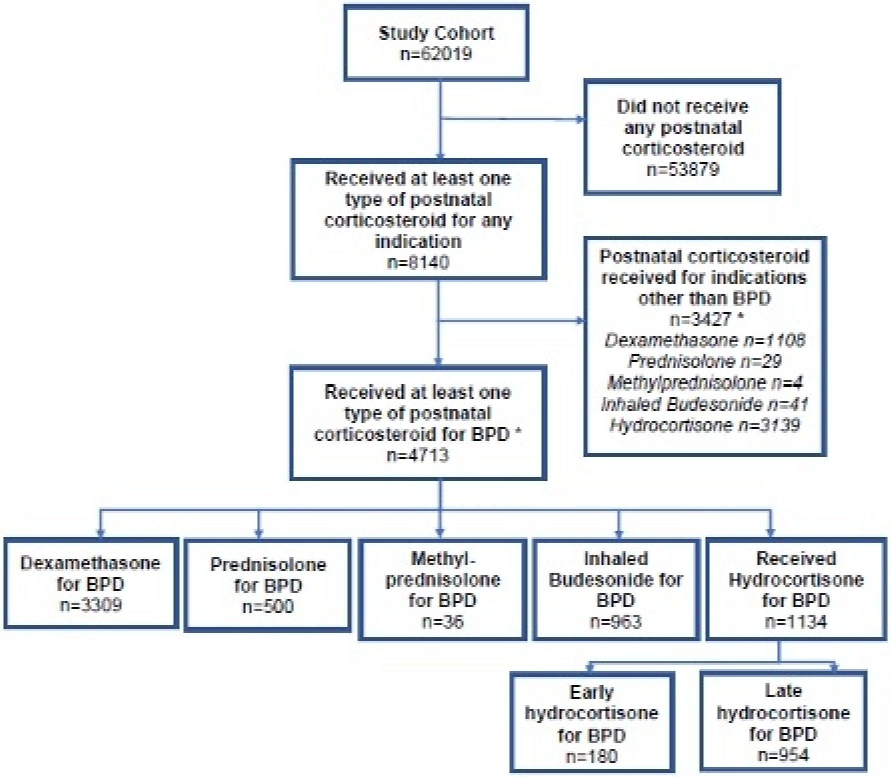

Figure 1. Flow chart of cohort who received postnatal steroids
*babies can have more than one postnatal corticosteroid type

157x147mm (72 x 72 DPI)

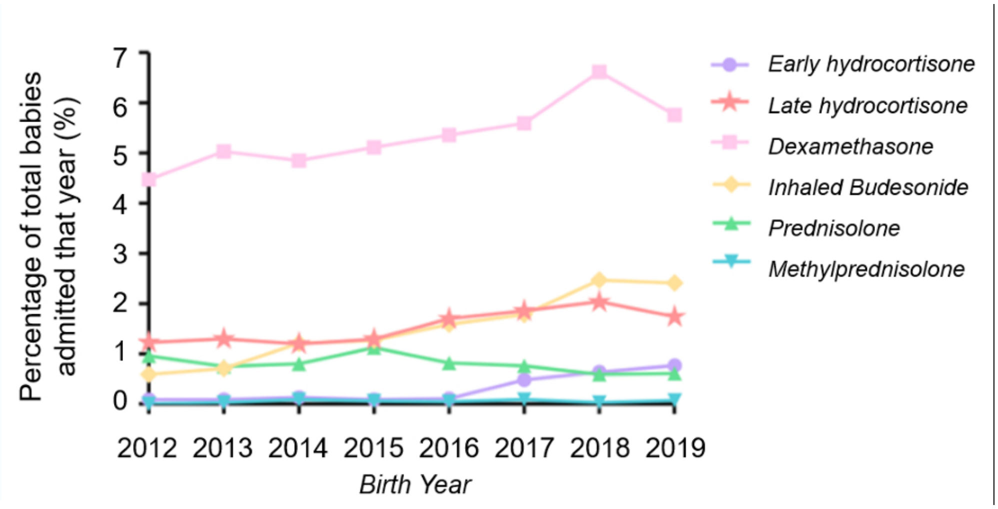

Figure 2. Graph showing the use of postnatal corticosteroids over time

159x79mm (150 x 150 DPI)

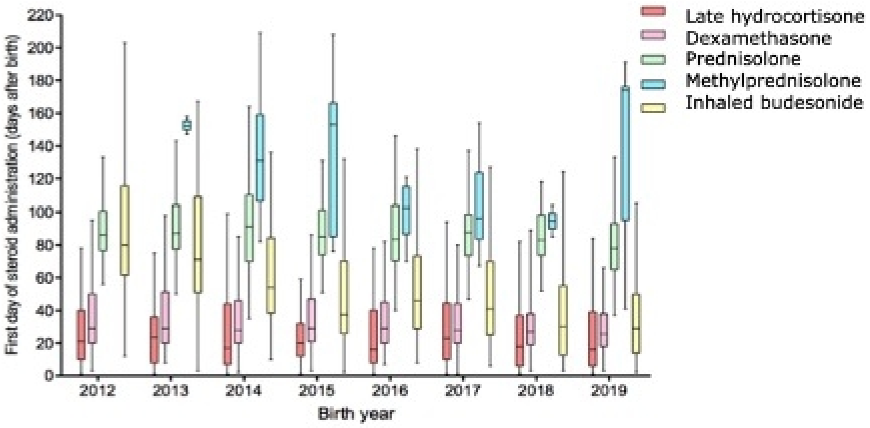

Figure 3. Boxplot showing the median, IQR and range of first day postnatal corticosteroid administration for BPD (grouped by postnatal corticosteroid type) in babies who were born at <32 weeks gestation between 2012-2019 and were admitted to neonatal units in England and Wales

159x83mm (72 x 72 DPI)

Supplementary file S1

Code	Diagnosis
15817	Neonatal intestinal perforation
15819	Neonatal ileum perforation
15821	Neonatal terminal ileum perforation
11010244	Intestinal perforation - not NEC
11217	Closure of jejunal perforation
11222	Closure of small intestine/ileal perforation

For peer review only

The RECORD statement – checklist of items, extended from the STROBE statement, that should be reported in observational studies using routinely collected health data.

	Item No.	STROBE items	Location in manuscript where items are reported	RECORD items	Location in manuscript where items are reported
**Title and abstract**					
	1	(a) Indicate the study's design with a commonly used term in the title or the abstract (b) Provide in the abstract an informative and balanced summary of what was done and what was found		RECORD 1.1: The type of data used should be specified in the title or abstract. When possible, the name of the databases used should be included.<br><br>RECORD 1.2: If applicable, the geographic region and timeframe within which the study took place should be reported in the title or abstract.<br><br>RECORD 1.3: If linkage between databases was conducted for the study, this should be clearly stated in the title or abstract.	page 2
**Introduction**					
Background rationale	2	Explain the scientific background and rationale for the investigation being reported			Page 4
Objectives	3	State specific objectives, including any prespecified hypotheses			Page 5
**Methods**					
Study Design	4	Present key elements of study design early in the paper			Page 5 and 6
Setting	5	Describe the setting, locations, and relevant dates, including periods of recruitment, exposure, follow-up, and data collection			Page 5

For peer review only

Participants	6	*(a) Cohort study* - Give the eligibility criteria, and the sources and methods of selection of participants. Describe methods of follow-up<br>*Case-control study* - Give the eligibility criteria, and the sources and methods of case ascertainment and control selection. Give the rationale for the choice of cases and controls<br>*Cross-sectional study* - Give the eligibility criteria, and the sources and methods of selection of participants<br><br>*(b) Cohort study* - For matched studies, give matching criteria and number of exposed and unexposed<br>*Case-control study* - For matched studies, give matching criteria and the number of controls per case		RECORD 6.1: The methods of study population selection (such as codes or algorithms used to identify subjects) should be listed in detail. If this is not possible, an explanation should be provided.<br><br>RECORD 6.2: Any validation studies of the codes or algorithms used to select the population should be referenced. If validation was conducted for this study and not published elsewhere, detailed methods and results should be provided.<br><br>RECORD 6.3: If the study involved linkage of databases, consider use of a flow diagram or other graphical display to demonstrate the data linkage process, including the number of individuals with linked data at each stage.	Page 5
Variables	7	Clearly define all outcomes, exposures, predictors, potential confounders, and effect modifiers. Give diagnostic criteria, if applicable.		RECORD 7.1: A complete list of codes and algorithms used to classify exposures, outcomes, confounders, and effect modifiers should be provided. If these cannot be reported, an explanation should be provided.	Page 6 and supplementary
Data sources/ measurement	8	For each variable of interest, give sources of data and details of methods of assessment (measurement).<br>Describe comparability of assessment methods if there is more than one group			Page 5

For peer review only

Bias	9	Describe any efforts to address potential sources of bias			Page 5
Study size	10	Explain how the study size was arrived at			Page 5
Quantitative variables	11	Explain how quantitative variables were handled in the analyses. If applicable, describe which groupings were chosen, and why			Page 6
Statistical methods	12	(a) Describe all statistical methods, including those used to control for confounding<br>(b) Describe any methods used to examine subgroups and interactions<br>(c) Explain how missing data were addressed<br>(d) *Cohort study* - If applicable, explain how loss to follow-up was addressed<br>*Case-control study* - If applicable, explain how matching of cases and controls was addressed<br>*Cross-sectional study* - If applicable, describe analytical methods taking account of sampling strategy<br>(e) Describe any sensitivity analyses			Page 6
Data access and cleaning methods		..		RECORD 12.1: Authors should describe the extent to which the investigators had access to the database population used to create the study population.	Page 5

For peer review only

				RECORD 12.2: Authors should provide information on the data cleaning methods used in the study.	
Linkage		..		RECORD 12.3: State whether the study included person-level, institutional-level, or other data linkage across two or more databases. The methods of linkage and methods of linkage quality evaluation should be provided.	N/A
**Results**					
Participants	13	(a) Report the numbers of individuals at each stage of the study (*e.g.*, numbers potentially eligible, examined for eligibility, confirmed eligible, included in the study, completing follow-up, and analysed)<br>(b) Give reasons for non-participation at each stage.<br>(c) Consider use of a flow diagram		RECORD 13.1: Describe in detail the selection of the persons included in the study (*i.e.,* study population selection) including filtering based on data quality, data availability and linkage. The selection of included persons can be described in the text and/or by means of the study flow diagram.	Page 6
Descriptive data	14	(a) Give characteristics of study participants (*e.g.*, demographic, clinical, social) and information on exposures and potential confounders<br>(b) Indicate the number of participants with missing data for each variable of interest<br>(c) *Cohort study* - summarise follow-up time (*e.g.*, average and total amount)			Page 7
Outcome data	15	*Cohort study* - Report numbers of outcome events or summary measures over time<br>*Case-control study* - Report numbers in each exposure			Page 8-10

For peer review only

For peer review only - http://bmjopen.bmj.com/site/about/guidelines.xhtml

		category, or summary measures of exposure<br>*Cross-sectional study* - Report numbers of outcome events or summary measures			
Main results	16	(a) Give unadjusted estimates and, if applicable, confounder-adjusted estimates and their precision (e.g., 95% confidence interval). Make clear which confounders were adjusted for and why they were included<br>(b) Report category boundaries when continuous variables were categorized<br>(c) If relevant, consider translating estimates of relative risk into absolute risk for a meaningful time period			Page 8-10
Other analyses	17	Report other analyses done—e.g., analyses of subgroups and interactions, and sensitivity analyses			
**Discussion**					
Key results	18	Summarise key results with reference to study objectives			Page 11
Limitations	19	Discuss limitations of the study, taking into account sources of potential bias or imprecision. Discuss both direction and magnitude of any potential bias		RECORD 19.1: Discuss the implications of using data that were not created or collected to answer the specific research question(s). Include discussion of misclassification bias, unmeasured confounding, missing data, and changing eligibility over time, as they pertain to the study being reported.	Page 11
Interpretation	20	Give a cautious overall interpretation of results considering objectives,			Page 11 and 12

		limitations, multiplicity of analyses, results from similar studies, and other relevant evidence			
Generalisability	21	Discuss the generalisability (external validity) of the study results			Page 11
**Other Information**					
Funding	22	Give the source of funding and the role of the funders for the present study and, if applicable, for the original study on which the present article is based			Page 3
Accessibility of protocol, raw data, and programming code		..		RECORD 22.1: Authors should provide information on how to access any supplemental information such as the study protocol, raw data, or programming code.	Supplementary

*Reference: Benchimol EI, Smeeth L, Guttmann A, Harron K, Moher D, Petersen I, Sørensen HT, von Elm E, Langan SM, the RECORD Working Committee. The REporting of studies Conducted using Observational Routinely-collected health Data (RECORD) Statement. *PLoS Medicine* 2015; in press.

*Checklist is protected under Creative Commons Attribution (CC BY) license.

For peer review only
